# Evolution of HIV-1 within untreated individuals and at the population scale in Uganda

**DOI:** 10.1371/journal.ppat.1007167

**Published:** 2018-07-27

**Authors:** Jayna Raghwani, Andrew D. Redd, Andrew F. Longosz, Chieh-Hsi Wu, David Serwadda, Craig Martens, Joseph Kagaayi, Nelson Sewankambo, Stephen F. Porcella, Mary K. Grabowski, Thomas C. Quinn, Michael A. Eller, Leigh Anne Eller, Fred Wabwire-Mangen, Merlin L. Robb, Christophe Fraser, Katrina A. Lythgoe

**Affiliations:** 1 Big Data Institute, Li Ka Shing Centre for Health Information and Discovery, Nuffield Department of Medicine, University of Oxford, Oxford, United Kingdom; 2 Department of Zoology, Peter Medawar Building, University of Oxford, Oxford, United Kingdom; 3 Laboratory of Immunoregulation, Division of Intramural Research, NIAID, NIH, Baltimore MD, United States of America; 4 Department of Medicine, Johns Hopkins Medical Institute, Johns Hopkins University, Baltimore MD, United States of America; 5 Department of Statistics, University of Oxford, Oxford, United Kingdom; 6 Rakai Health Sciences Program, Kalisizo, Uganda; 7 School of Public Health, Makerere University, Kampala, Uganda; 8 Genomics Unit, RTS, RTB, Rocky Mountain Laboratories, Division of Intramural Research, NIAID, NIH, Hamilton MT, United States of America; 9 School of Medicine, Makerere University, Kampala, Uganda; 10 Department of Pathology, Johns Hopkins Medical Institute, Johns Hopkins University, Baltimore, MD, United States of America; 11 U.S. Military HIV Research Program, Walter Reed Army Institute of Research, Silver Spring, MD, United States of America; 12 Henry M. Jackson Foundation for the Advancement of Military Medicine, Bethesda, MD, United States of America; Katholieke Unversiteit Leuven, BELGIUM

## Abstract

HIV-1 undergoes multiple rounds of error-prone replication between transmission events, resulting in diverse viral populations within and among individuals. In addition, the virus experiences different selective pressures at multiple levels: during the course of infection, at transmission, and among individuals. Disentangling how these evolutionary forces shape the evolution of the virus at the population scale is important for understanding pathogenesis, how drug- and immune-escape variants are likely to spread in populations, and the development of preventive vaccines. To address this, we deep-sequenced two regions of the HIV-1 genome (p24 and gp41) from 34 longitudinally-sampled untreated individuals from Rakai District in Uganda, infected with subtypes A, D, and inter-subtype recombinants. This dataset substantially increases the availability of HIV-1 sequence data that spans multiple years of untreated infection, in particular for different geographical regions and viral subtypes. In line with previous studies, we estimated an approximately five-fold faster rate of evolution at the within-host compared to the population scale for both synonymous and nonsynonymous substitutions, and for all subtypes. We determined the extent to which this mismatch in evolutionary rates can be explained by the evolution of the virus towards population-level consensus, or the transmission of viruses similar to those that establish infection within individuals. Our findings indicate that both processes are likely to be important.

## Author summary

The speed at which HIV-1 evolves within individuals and across epidemics is substantially different. Identifying the mechanisms shaping this phenomenon has important implications for understanding disease severity and transmission of HIV-1, especially when considering the spread of viruses that are resistant to drugs or natural host defenses in the population. In this study, we analyze newly generated HIV-1 sequences sampled from 34 individuals living in Uganda over multiple years, together with publicly available HIV-1 sequences from Uganda at the population scale. Our findings indicate that HIV-1 evolves around five times faster within individuals compared to the population scale. We demonstrate that there are likely two key processes driving the difference in HIV-1 evolutionary rate at the within individual and population scales. Specifically, we find support for 1) selection against variants in the within-host viral population, which were acquired in the donor because they were beneficial in that individual, and 2) preferential transmission of variants that are similar to those that initiated the infection.

## Introduction

Infection by HIV-1 is lifelong, and characterized by ongoing viral replication, and consequently the virus can undergo hundreds of rounds of replication between transmission events. This, combined with error-prone viral replication during reverse transcription, means that the viruses an individual transmits to a recipient are unlikely to be identical to those that initially infected them. A firm understanding of how evolution proceeds during the course of infection within an individual, and how this corresponds to evolution of the virus at the population scale, is therefore required to understand how selection acts at the point of transmission. This is important for vaccine design, and understanding how the virus evolves at the epidemiological scale.

To understand the natural history of within-host HIV-1 infection, historical samples from untreated individuals are needed. The few datasets that exist, where multiple (>2) within-host sequenced samples are available, including from early infection, and spanning years rather than months, include nine subtype B individuals from North America [1], ten individuals from Europe, where eight were infected with subtype B, and two were infected with subtypes C and AE, respectively [2,3], and four female individuals from North America infected with subtype B [4]. Here, we add considerably to this small but important body of data by presenting longitudinal deep-sequencing data from 34 untreated individuals living in Rakai, Uganda, representing infection with pure subtypes A and D, and a variety of inter-subtype recombinants, thus expanding both the geographical regions and the viral subtypes for which data are available. As well as undertaking an evolutionary analysis of the within-host sequence data, we also determined the rate of evolution of subtypes A, C, and D at the population scale within Uganda, during approximately the same time period. Interestingly, we found that the nonsynonymous substitution rate in the gp41 region of *env* is twice as fast for subtype C compared to other subtypes.

An indication that evolution at the population scale does not merely represent a continuation of directional within-host evolution, with repeated bottlenecks at the point of transmission, is the observation that HIV-1 evolves about two to six times faster within hosts than at the population scale [5–8] for both synonymous and nonsynonymous mutations [9–11]. Although here we examined different subtypes and gene regions to previous studies, our analysis confirms a similar mismatch in evolutionary rates.

Three alternative hypotheses for the mismatch in HIV-1 evolutionary rates have been proposed [11]: The first, called ‘stage-specific selection’, suggests that transmission tends to occur early in infection, when within-host evolution is also suggested to be slow, resulting in slower than expected evolution at the population level [12]. Stage-specific selection is supported by the observation that the rate of evolution at the population level was found to be slower in populations where transmission probably occurs earlier during infection, such as among men-who-have-sex-with-men (MSM) [12]. Although still subject to debate, stage-specific selection is unlikely since cellular and antibody driven escape mutations have been shown to develop rapidly during early infection [13–17], and because stage-specific selection is expected to lead to a greater mismatch for nonsynonymous rather than synonymous substitutions; a pattern that we do not see [11]. Moreover, a more recent study found a faster rate of evolution in MSM than in heterosexual transmission chains [18], suggesting other factors likely explain differing rates of evolution among different groups.

The second hypothesis, called ‘adapt and revert’, suggests that within-host evolution is dominated by the ‘reversion’ of mutations that were advantageous in the source host but detrimental in the recipient host [2,11,19–22]. This hypothesis is supported by the observation that within-host evolution is biased towards the accumulation of mutations towards the consensus at the population level [2]. The use of the term of reversion here is contentious because it implies adaptive evolution is going backwards, undoing what was selected for in a previous individual. However, at an individual level, adaptive evolution always goes forwards, thereby selecting for fitter genotypes. It is only when we step back to consider forward within-host evolution in the context of the viruses circulating at the population level that reversion is observed.

The final hypothesis suggests that during the course of infection viral lineages that resemble the virus that initiated the infection are maintained, and that these ‘founder-like’ viruses are preferentially transmitted. In this scenario, the cycling of viral lineages through the HIV-1 reservoir maintains these founder-like viruses within the population [6,11,23–27], and therefore this process is referred to as ‘store and retrieve’. This hypothesis is supported by the analysis of HIV transmission chains, in which slower evolving lineages are transmitted [7]; studies of discordant couples showing evidence for the transmission of founder-like viruses [28,29]; variation in the rate of evolution along different within-host lineages [5,30]; and the observation that viruses founding new infections are adapted to early infection, yet under-represented in donor populations [17,31].

Adapt and revert and store and retrieve have largely been presented as either/or scenarios, but these processes need not be mutually exclusive [11]. Moreover, recombination and differing selection pressures in different genomic regions, means their contributions might not be homogeneous across the genome [2,26]. Using longitudinal deep-sequenced data from 34 HIV-1 seroconverters from the Rakai District in Uganda, we tested specific predictions of the adapt and revert and store and retrieve hypotheses, in an effort to quantify their relative contributions. First, we tested whether synonymous and/or nonsynonymous mutations show a strong bias in substitutions towards the population consensus, as predicted by ‘adapt and revert’ [2]. Second, we tested whether a sufficient number of founder-like viruses persist as infection progresses for their preferential transmission to explain the mismatch in phylogenetic rates for synonymous and/or nonsynonymous mutations, as required for store and retrieve [11]. Rather than being a case of either/or, our data suggest that both processes are likely to be important in explaining the mismatch in evolutionary rates. Moreover, our analysis supports previous work that purifying selection can explain why rates of viral evolution decline as the timescales over which they are measured increase from decades to millennia [32–36].

## Results

### Study population

Thirty four HIV-1 seroconverters from the Rakai District in Uganda, previously found not to be superinfected with HIV-1 [37] using deep-sequencing (Roche 454, Pleasanton CA) were selected for further analysis based on sample availability (Table 1). In the previous superinfection screen, early and late samples were sequenced at the p24 region of *gag* and the gp41 region of *env* [37]. We sequenced serum samples from three additional time points after HIV-1 seroconversion and prior to initiation of ART (anti-retroviral therapy) using identical methods. These data were combined with the previous sequence data for all subsequent analyses, resulting in longitudinal deep-sequencing data for a 390 base pair (bp) region of p24 and a 324 bp region of gp41.

**Table 1:** Summary information for the 34 individuals and corresponding samples analysed in this study.

Individual	Gender	Age at seroconversion	p24 subtype	gp41 subtype	Log SPVL^a^	First sample^b^ (days)	Last sample^b^ (days)	Number p24 samples	Number gp41 samples
i1	F	46	D	D	5.03	175	1268	5	5
i2	F	31	D	D	4.71	219	2276	5	5
i3	F	28	D	D	4.86	214	2249	2	4
i4	F	30	D	D	4.86	225	1190	5	5
i5	F	31	A	A	4.08	213	1826	5	5
i6	M	40	C	A		237	672	5	5
i7	F	26	D	D	5.69	202	2268	5	5
i8	F	32	D	D	4.02	218	2946	5	5
i9	M	38	D	D	4.53	198	910	4	4
i10	M	23	D	A	3.17	173	2110	5	5
i11	M	24	A	A	5.81	363	1776	4	4
i12	M	31	D	D	5.12	291	1734	5	4
i13	M	42	D	D	4.28	234	1757	5	5
i14	F	24	D	D	5.29	425	1688	5	5
i15	F	18	A	A	3.66	204	2042	5	5
i16	M	31	D	A	4.96	228	666	5	5
i17	F	21	D	D	3.96	191	1919	5	4
i18	F	18	A	A	5.23	150	1040	5	5
i19	F	20	D	D	5.24	228	679	4	4
i20	F	35	D	D		198	910	5	5
i21	M	23	A	D	4.96	246	1724	5	5
i22	M	37	D	D	3.71	240	674	5	5
i23	F	30	D	D	4.22	230	1863	5	5
i24	F	21	D	D	5.02	226	1207	5	5
i25	F	35	A	A	5.35	226	1806	5	5
i26	F	36	D	D	5.1	235	1144	3	5
i27	F	24	D	C	4.13	239	2365	5	5
i28	F	22	D	D	5.6	234	654	5	5
i29	F	29	D	D	4.4	168	1246	5	5
i30	M	22	A	A	5.12	187	1927	4	4
i31	F	25	D	A	4.24	174	2096	5	5
i32	M	22	A	A	4.37	204	1624	5	5
i33	F	30	D	D	4.61	208	1652	5	5
i34	M	23	A	D	4.4	193	927	5	5

^a^ Set-point viral load

^b^Estimated number of days since seroconversion for the first and last sequenced samples. The seroconversion date was estimated as the mid-date between the last negative and first positive samples. For all individuals except i11, the first sequenced sample corresponds to the first positive sample, giving a window +/- the number of days shown in the first sample column. For i11, the window is +/- 340 days.

### Summary of within-host molecular evolutionary patterns

**Fig 1:**
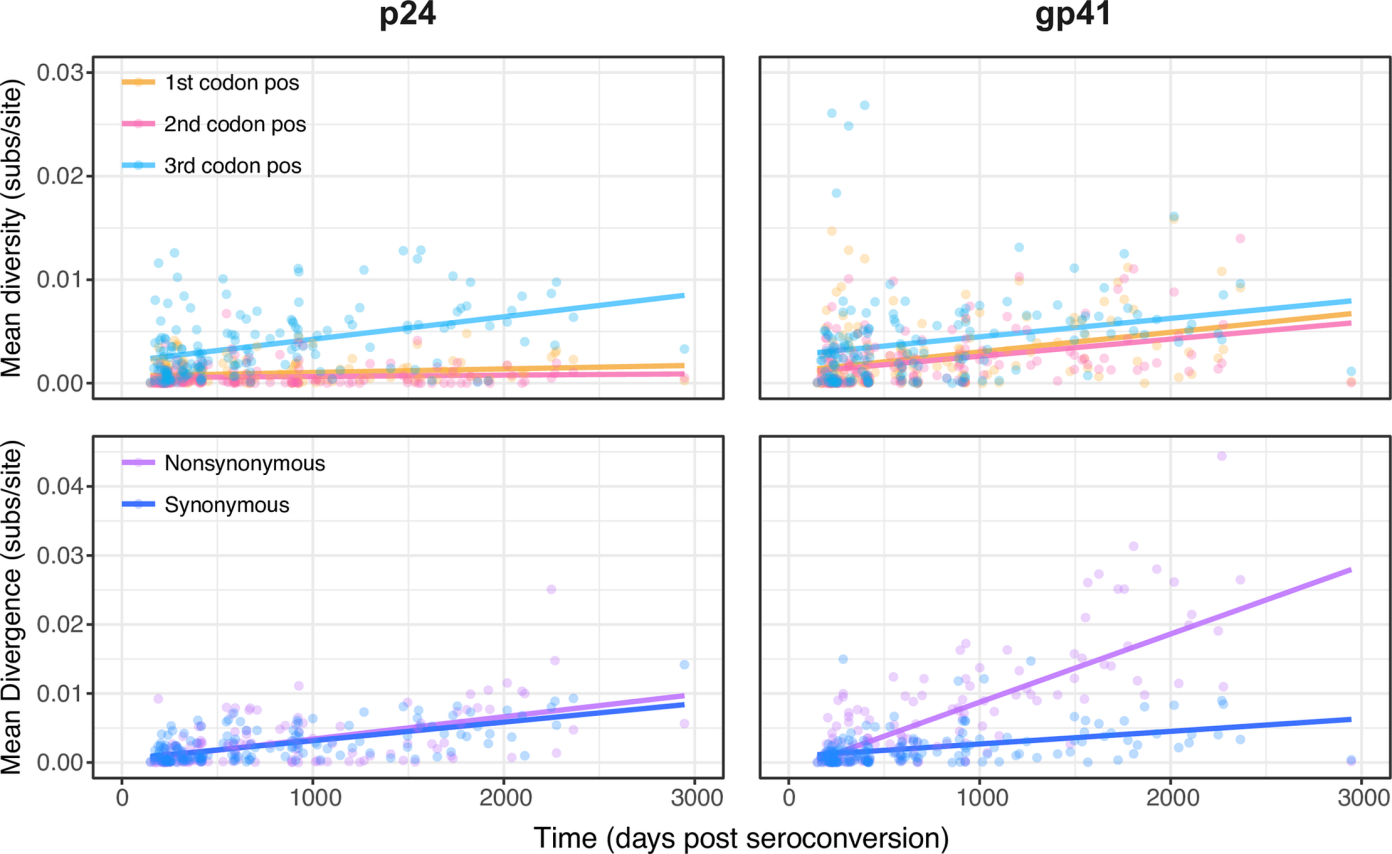
Diversity and divergence over time for 34 individuals for both p24 and gp41. Top Row: Mean pairwise diversity at first, second, and third codon positions over time for individuals (represented in yellow, pink, and light blue, respectively). The average change in mean pairwise diversity over time was inferred by linear regression. Bottom Row: Mean nonsynonymous (purple) and synonymous divergence (blue) over time for individuals.

Fig 1 illustrates mean diversity (at 1^st^, 2^nd^ and 3^rd^ codon positions) and divergence (for synonymous and nonsynonymous changes) over time among the 34 individuals for gp41 and p24. We fitted a linear regression to the diversity and divergence estimates over time among the 34 individuals. Diversity at third codon positions accumulated at similar rates in p24 and gp41, but diversity at the first and second positions accumulated much more slowly for p24 compared to gp41 (Fig 1). This is consistent with stronger purifying selection acting upon p24 and stronger diversifying selection acting upon gp41. This is further supported by the divergence patterns. In p24, nonsynonymous and synonymous substitutions accumulated at approximately similar rates (Fig 1; bottom left panel). In contrast, for gp41 notably greater nonsynonymous divergence than synonymous divergence was observed (Fig 1; bottom right panel). We further examined the diversity and divergence patterns by excluding nine individuals, which were likely infected with multiple viral variants (defined as > = 0.02 mean pairwise diversity across all sites in the p24 gene region at the first time point). This may not identify all individuals where multiple viral variants were transmitted, particularly if these variants are very similar or where one variant has been lost. Nevertheless, no discernible impact on mean diversity and divergence trends was observed when these individuals were excluded (S1 Fig).

Although diversity and divergence tended to increase as infection progressed, we observed considerable variation among individuals, with diversity notably not always increasing between subsequent time points (S2 Fig). To some extent, this reflects stochastic error associated with short gene regions and the 454-sequencing method (S2 Fig). In particular, biases in genome amplification during PCR are likely to result in diversity being underestimated. However, within-host evolutionary dynamics probably also has an important role. One individual (i24) had very high diversity in the gp41 gene region at the third codon position at the first sampling time point, which subsequently declined over time (S2A Fig; bottom right panel). Inspection of the maximum clade credibility trees (summary of the posterior tree distribution from BEAST) indicates this individual was infected by at least two different viral variants, which were very similar in p24, but very different in gp41 (S3 Fig). This is reflected in the low posterior support for the two infecting lineages in the p24 tree, but high posterior support (0.82–1.0) for multiple infecting lineages in the gp41 tree, which were maintained throughout infection (S3 Fig). The fall in diversity at the third codon position (S2 Fig) between the penultimate and last time points for i24 corresponds to one of the lineages predominating among the sampled viruses (S3 Fig). This probably reflects ongoing within-host competition between the two lineages, with eventually one lineage prevailing. A similar pattern of falling diversity is seen in the p24 gene region of i17, where a fall in diversity at the last sampled time point (1919 days) coincides with the loss of one of the within-host lineages (S4 Fig). Given sequence similarity at the first time point, the multiple lineages observed in i17 probably emerged during infection rather than due to coinfection. In the gp41 region of this individual we also see a drop in diversity between days 828 and 923. This possibly reflects the complex within-host evolutionary dynamics inferred by the MCC tree of this individual (S4 Fig), although since this individual had a relatively low SPVL (Table 1), amplification errors during sequencing could affect the estimates of diversity.

### Within- and between-host evolutionary rates

**Fig 2:**
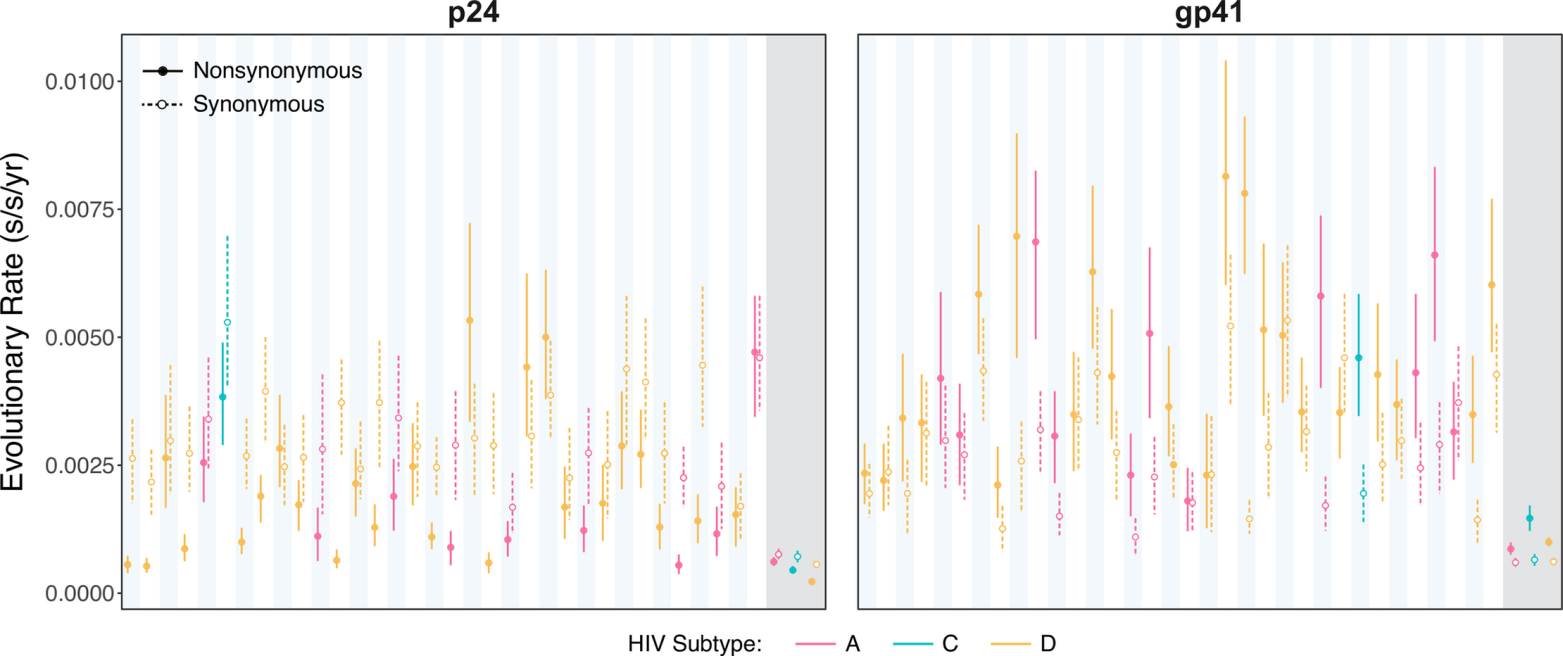
Evolutionary rates for p24 and gp41 at the within- and between-hosts scales measured in substitutions per site per year (subs/site/year). The within-host nonsynonymous (filled circles) and synonymous (open circles) substitution rates were estimated for p24 and gp41 for all 34 individuals, which are ordered from left to right (subtype A, pink; subtype C, green; subtype D, yellow). Blue and white columns correspond to the individual estimates. The estimates in the grey background indicate the between-host substitution rates for subtypes A, C, and D. The vertical lines represent the 95% credible intervals (solid lines, nonsynonymous; dashed lines, synonymous).

Next we looked at absolute nonsynonymous and synonymous substitution rates at the within-host level. Estimated rates of within-host viral evolution varied considerably among individuals (Fig 2), and were consistent with previous measures of within-host rates of viral evolution for subtype B infected individuals in *gag* [4] and the gp120 region of *env* [4–6]. The comparatively large uncertainty in the estimates of within-host evolutionary rates are most likely due to the short gene regions analyzed and lower rates of evolution compared to gp120. Regardless, a significant mismatch was observed between the within- and between-host evolutionary rates in both gene regions for subtypes A, C, and D (Fig 2). Note that for recombinant infections, individuals have different subtypes at each of the two regions (Table 1). The mean ratios of the within- and between-host rates ranged from 3.0 to 8.8, and were similar for nonsynonymous and synonymous substitutions, and did not differ by gene region or subtype (S1 Table), although some of these estimates were associated with large uncertainties (standard deviation ranged from 1.1 to 5.9). It has previously been argued that higher rates of nonsynonymous and synonymous substitution at the within-host level compared to the between-host level is compatible with store and retrieve, but not stage-specific selection or adapt and revert [11], since under the latter two hypotheses a mismatch is only expected for nonsynonymous substitutions. However, this assumes that synonymous substitutions experience lower levels of selection compared to nonsynonymous substitutions, which might not always be the case due to their effects on RNA secondary structure [38].

In line with the patterns observed for divergence over time, a greater nonsynonymous substitution rate was noted for gp41 than for p24 among the different subtypes, whereas synonymous substitution rates were comparable (S5 Fig). These observations are consistent with gp41 being subjected to stronger diversifying selection than p24, together with p24 undergoing comparatively greater purifying selection. We see similar patterns in the between-host evolutionary rates (Fig 2). Notably, subtype C was characterized by the highest overall substitution rate in gp41 (~0.002 subs/site/year), which appears to be driven by a comparatively higher nonsynonymous substitution rate (Fig 2 and S1 Table). This elevated rate for subtype C is in line with a previous study, but where substitution rates were estimated from whole-genome sequences that were sampled from a broader geographic distribution [39].

### Sensitivity analysis

To corroborate the within-host evolutionary estimates using the renaissance counting method and hierarchical phylogenetic model, we performed three sets of auxiliary analyses in BEAST for a subset of ten individuals consisting of five pure subtype A and five pure subtype D infections, i.e. for a given HIV-1 infection, p24 and gp41 gene regions corresponded to the same subtype. Furthermore, based on mean diversity at the first time point in the p24 gene region, these individuals were unlikely to have been infected by multiple, genetically distinct strains. The results from the sensitivity analyses are briefly discussed here, but a full description can be found in S1 Text. First, we found a strong correspondence in the evolutionary rates for the ten individuals from the original analysis (based on 34 individuals) and the new analysis, which was based on ten individuals with pure subtype infections (S6 Fig). This strongly suggests that individuals infected with multiple variants have not impacted the evolutionary rate estimates for individuals infected with single viral variants. Next, we estimated evolutionary rates for the subset of individuals using a full codon substitution model [40], which were in good agreement with estimates from the renaissance counting method (S7 Fig). Finally, we examined the robustness of the evolutionary rate estimates for the ten individuals to different hyperpriors in the clock hierarchical phylogenetic model (see S1 Text for more details). The evolutionary rate estimates were found to be very similar across different hyperpriors (S8 Fig), strongly suggesting that the posterior estimates of the within-host evolutionary rates in Fig 2 are mostly informed by the sequence data and are robust to the choice of hyperprior in the hierarchical phylogenetic model.

### Link between within-host rates of evolution and set-point viral load

In a previous analysis comparing within-host rates of evolution with set-point viral load (SPVL), Lemey *et al*. observed a significant correlation between the synonymous substitution rate of the C2V5 region along the backbone branches (ancestral internal branches in the phylogeny that have given rise to the most recently sampled sequences) and the rate of disease progression [10]. Specifically, individuals with slower disease progression had lower rates of synonymous substitution than individuals with faster disease progression. Since SPVL is a strong predictor of disease progression in HIV-1 infection, we examined the correlation between the within-host evolutionary rate and SPVL to determine whether a similar relationship exists between synonymous substitution rate and disease progression in the Rakai cohort. In contrast to Lemey *et al*., we found no significant correlation between the mean absolute synonymous or non-synonymous substitution rate (on either external, internal, or backbone branches) and SPVL (S9 Fig; S3 Table). We also tested for a subtype-specific effect, but again, we did not find a significant correlation. The lack of a significant correlation observed here could be because there is no link between substitution rate and SPVL among the Rakai individuals, or because of uncertainty in the substitution rate and/or SPVL estimates. In particular, the p24 and gp41 gene regions have low rates of evolution compared to the C2V5 region, which is typically associated with an order of magnitude higher rate of evolution (see Fig 3 in [2]).

**Fig 3:**
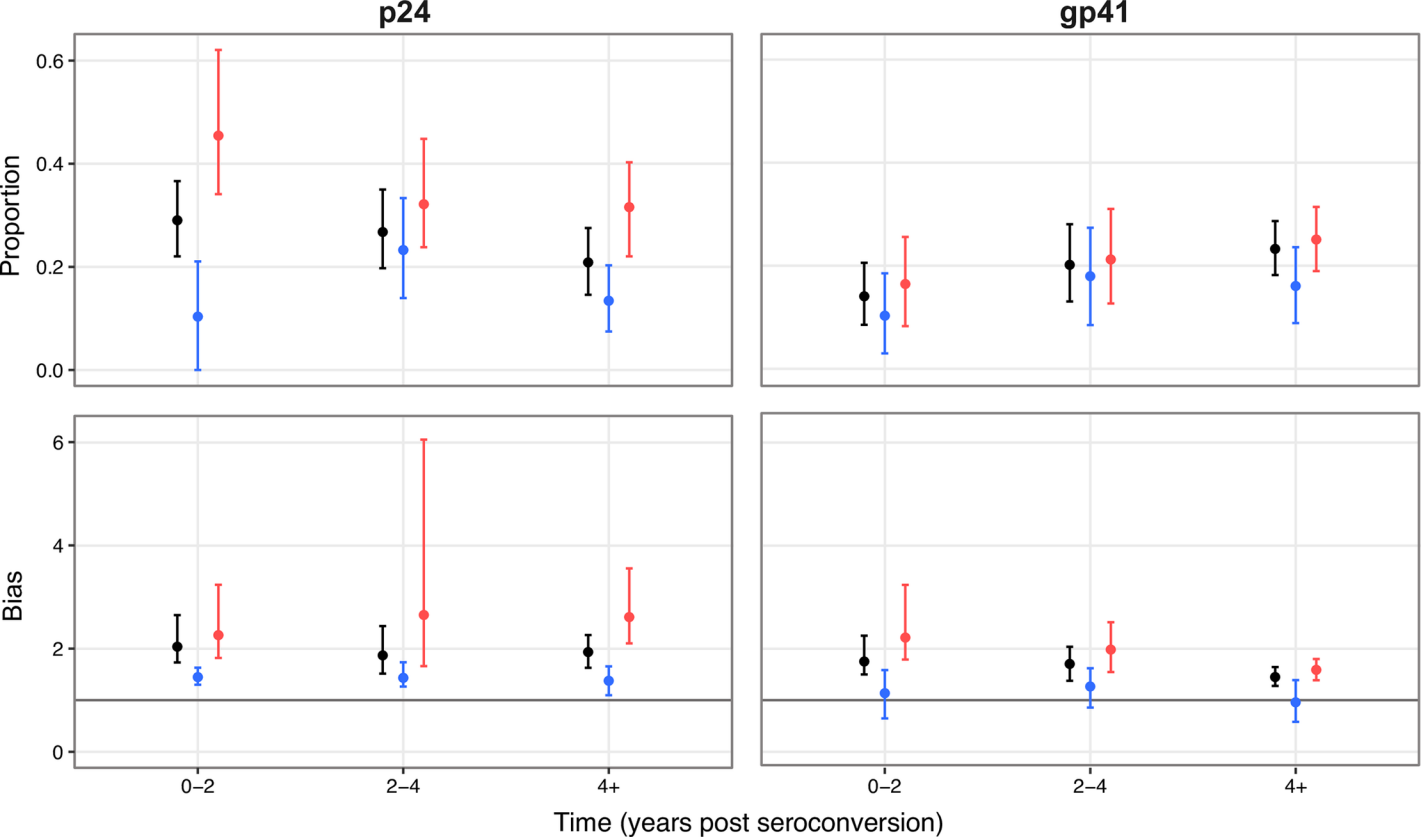
Changes towards population consensus. Top Row: The proportion of polymorphic sites where a mutant allele represents a change towards the (subtype-specific) population consensus. Bottom Row: The bias in changes towards population consensus. This is the ratio of the proportion of changes that are towards (subtype-specific) population consensus, compared to the expected proportion in the absence of selection, measured at polymorphic sites and with an assumed mutational transition to transversion ratio of two. A bias of 1 means the proportion of changes that are towards population consensus matches the expectation. The error bars give the 5 and 95 percentiles from 10,000 bootstraps of the individual data. Black, all changes; Blue, synonymous changes; Red, nonsynonymous changes.

### Evolution towards population consensus

A prediction of adapt and revert is that, of sites where evolutionary change is observed, the accumulation of mutations should be biased towards the population consensus as infection progresses [2]. Limiting our analysis to sites within individuals where the most common (founder) allele at the first time point was at or close to fixation (frequency >0.99), we defined polymorphic sites as those where a different (mutant) allele had reached a frequency of >0.1 at some point during the sampling period. Across all individuals, between 10 and 30 percent of the mutant alleles observed at polymorphic sites represented changes towards population consensus in p24 and gp41 (Fig 3, top row). Moreover, there was a strong bias, with changes towards population consensus at polymorphic sites occurring approximately 1.5 to 2 times more often than expected in the absence of selection (Fig 3, bottom row). Here, we assumed a mutational transition to transversion ratio of two, representing a biochemical preference for transitions. The broad pattern remains the same if we do not assume a preference for transitions, although the calculated bias is higher (S10 Fig). Taken together, these observations can explain a substantial mismatch in evolutionary rates, in broad agreement with a similar analysis using deep-sequencing data from nine European individuals [2]. In addition, if viruses that are more similar to the population consensus are preferentially transmitted [41], an even higher mismatch in evolutionary rates could be explained.

We next considered nonsynonymous and synonymous changes separately. A high proportion of nonsynonymous changes (mean proportion of 0.45 and 0.17, respectively, for p24 and gp41, at 0–2 years post seroconversion) were towards population consensus (Fig 3, top row). Furthermore, at polymorphic sites, these nonsynonymous changes were associated with a strong bias, such that changes towards population consensus occurred twice as often as expected (Fig 3, bottom row). Considering all sites close to fixation at the first sampling time point (not just polymorphic sites), and assuming mutations are equally likely at all sites, nonsynonymous changes continued to show a strong bias towards population consensus (S10 Fig). Together, these observations provide strong evidence for the role of adapt and revert for nonsynonymous substitutions, particularly in p24, which is consistent with stronger functional constraints being present in p24 compared to gp41.

A reasonably high proportion of synonymous changes were also towards population consensus (mean proportion of 0.10 and 0.11, respectively, for p24 and gp41, at 0–2 years post seroconversion; Fig 3, top row). This was accompanied by a small bias at polymorphic sites in p24, but there was no detectable bias in gp41 (Fig 3, bottom row). The absence of a strong bias at polymorphic sites suggests adapt and revert contributes little to the mismatch in evolution rates for synonymous changes when comparing rates at the within-host and population scales. Considering all sites, however, a bias towards population consensus was observed for synonymous changes in both gene regions, although this bias was much smaller than for nonsynonymous changes (S10 and S11 Figs). In other words, of all the changes that could occur (the vast majority of which will be away from population consensus, since most sites are at population consensus), and assuming mutation is equally likely at all sites, synonymous changes towards population consensus are much more likely than would be expected by chance. The detection of this bias for synonymous changes, when all sites are considered, provides evidence that a high proportion of synonymous changes are non-neutral, because, for example, they affect RNA secondary structure [38]. This is consistent with the observation that across the whole subtype B HIV-1 genome, synonymous mutations that occur away from population consensus during infection are often weakly deleterious, but with a significant proportion (~10% outside of *env*) being highly deleterious [42]. Evolution towards population consensus for synonymous changes, when all sites are considered, therefore most likely represents weak purifying selection, possibly exacerbated by the transmission of viruses harboring slightly deleterious mutations due to tight bottlenecks at transmission [33].

### Founder-like virus persists during the course of infection

Assuming virus is not transmitted directly from the reservoir, a key prediction of store and retrieve is that founder-like viruses should be circulating in the viral population at sufficient frequencies, with a mismatch in evolutionary rates occurring if these founder-like viruses are preferentially transmitted. We determined how ‘founder-like’ a virus is within an individual by the number of mutations, *d*, the virus differs from the consensus virus(es) circulating at the first sampled time point. Thus, we are essentially using a small portion of the genome (p24 or gp41) as a surrogate for the whole genome. We next assumed that each viral sequence has a transmission fitness *w*_*d*_ = *e*^−*α d*^, where *α* determines how rapidly transmissibility declines as sequences evolve away from the first time point consensus sequence(s), under the assumption that *d* is representative of how founder-like the whole genome is. The predicted contribution to the mismatch in evolutionary rates of an individual at a given sampling time point is then given by the mean value of *d* in the viral population at that time point, divided by the expected mean value of *d* in the transmitted viral population (see Methods).

**Fig 4:**
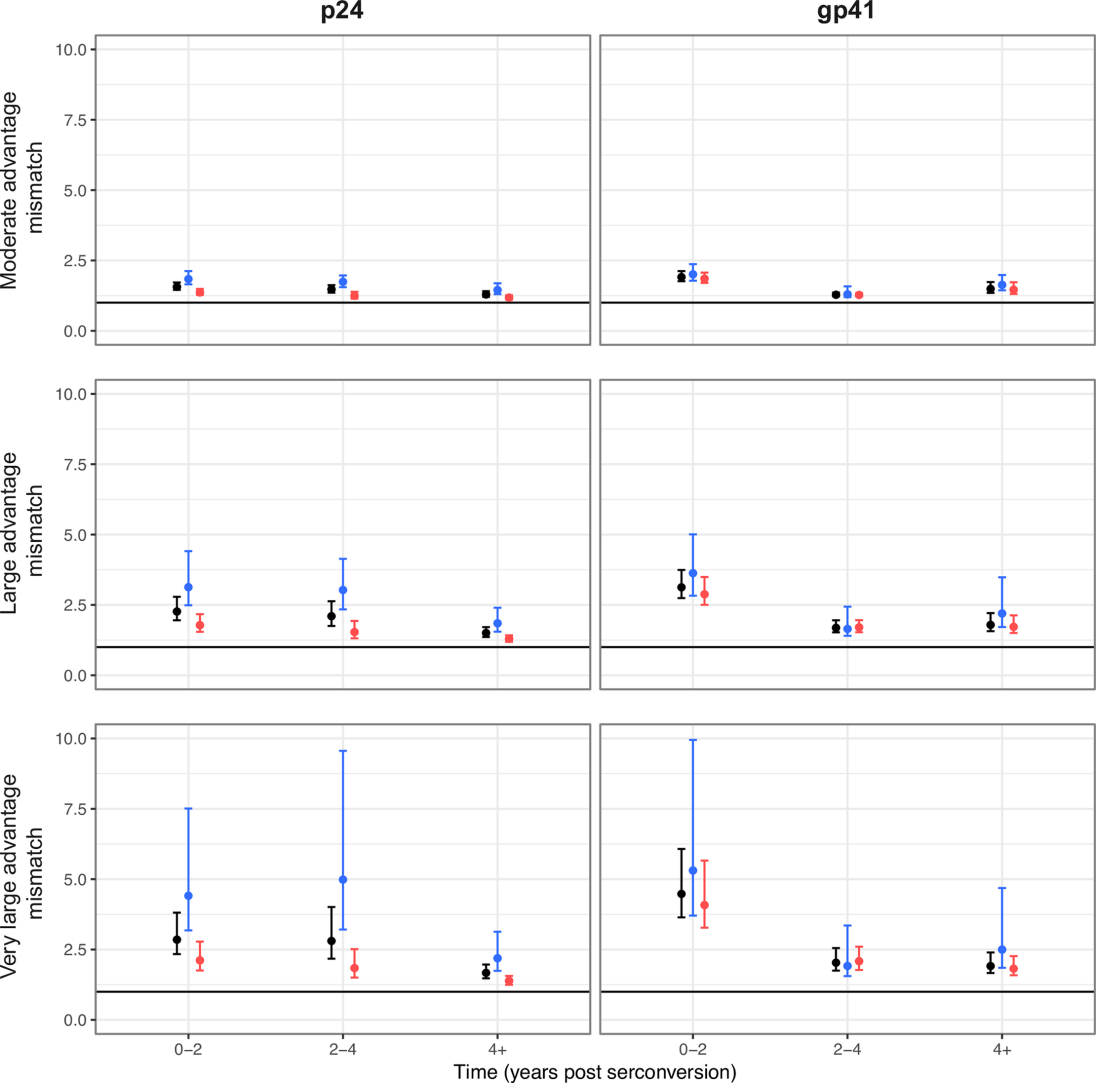
Contribution to the mismatch in evolutionary rates if founder-like virus has a transmission advantage. Each point represents the mean contribution of the 34 individuals to the mismatch in phylogenetic rates if transmission occurs during the given time period, and where each viral sequence has a transmission fitness *w*_*d*_ = *e*^−*α d*^. The contribution to the mismatch for each sampling time point for each individual was calculated as the ratio of the mean number of mutations from the founder population, *μ*, to the expected mean distance of transmitted virus from the founder population, *μT*, giving *m* = *μ*/*μT*. A mismatch of 1 therefore indicates the case where the within- and between-host rates of evolution are expected to be the same. We show results for a moderate (*α* = 1), large (*α* = 2), and very large (*α* = 3) transmission advantage. The error bars give the 5 and 95 percentiles from bootstrapping over the individuals 100,000 times. Black, all mutations; Blue, only synonymous mutations are considered when calculating the expected mismatch; Red, only nonsynonymous mutations are considered when calculating the expected mismatch (see Methods).

For both gene regions, a large mismatch in evolutionary rates can be explained if founder-like viruses have a strong transmission advantage (α ~ 2), and if transmission tends to occur during the first few years of infection (Fig 4). This pattern remains if we remove individuals likely infected by multiple variants (S12 Fig). As infection progresses, transmitted viruses are predicted to contribute less to a mismatch in evolutionary rates due to the gradual loss of founder-like viruses. A mismatch is still predicted if the data are partitioned between synonymous and nonsynonymous mutations (assuming selection at transmission is determined by the total number of mutations), although the predicted mismatch is generally less for nonsynonymous mutations. This is presumably because nonsynonymous mutations are subject to stronger within-host selection, and therefore founder-like variants are less likely to be preserved as infection progresses.

When interpreting these results, it is important to acknowledge the role of recombination, which for within-host viral populations has been shown to limit linkage disequilibrium to about 100–200 bps [2]. However, if founder-like viral lineages are maintained during infection (because they have spent a long time in the reservoir where neither error-prone replication nor recombination occur) linkage across much longer regions, and possibly the whole genome, is expected for these lineages [26]. Because we do not have linkage information between sequences in p24 and gp41, we were unable to determine whether viruses that harbor founder-like p24 sequences also harbor founder-like gp41 sequences, as would be expected if the preferential transmission of founder-like viruses explains the mismatch observed in both regions, and more generally the mismatch observed across the whole genome [8]. Thus, although our observations are consistent with store and retrieve, due to short read lengths (390 bp for p24 and 324 bp for gp41), and relatively low rates of evolution for these two gene regions, we have insufficient power to test whether these founder-like viral variants are maintained because of cycling of lineages through the viral reservoir, or simply due to the stochastic nature by which mutations are accumulated along lineages in these two short gene regions. To resolve this question, longitudinal, long-read deep-sequencing data is needed.

## Discussion

Using cryopreserved samples from individuals longitudinally sampled before the availability of universal treatment in the Rakai District of Uganda, we have substantially increased the number of individuals for which deep-sequenced data is available for HIV-1 during the course of untreated infection. The main subtypes represented in this HIV-1 cohort are A and D, rather than subtype B, which predominates in the more frequently studied European and North American cohorts. As well as analyzing this within-host sequence data, we also utilized publicly available population consensus sequences from Uganda, enabling us to directly compare rates of viral evolution at both the within-individual and population scales in the same population and for the same regions of the genome.

It is notable that the virus evolves approximately three to nine times faster at the within-host than at the population scale. This pattern was observed for all three subtypes in both gene regions and for nonsynonymous and synonymous substitutions. These estimates are consistent with previous estimates for nine subtype B infected individuals for the gp120 region of *env* [1,5,6], and for a subtype B infected individual measured across the whole genome [8]. Together with the observation that within-host viral lineages leading to transmission events evolve approximately half as fast as other lineages [7], these findings build a consistent picture of different rates of evolution for HIV-1 within- and between-hosts, for all of the subtypes that have been analyzed, and across the whole genome. Intriguingly, similar mismatches in evolutionary rates are observed for HIV-2, Hepatitis B, and Hepatitis C viruses [27,43–47], leading us to speculate that such mismatches are a general feature of rapidly evolving chronic viral infections in humans.

We tested specific predictions of two of the mechanisms that have been implicated as contributing to the mismatch in evolutionary rates in HIV-1: adapt and revert and store and retrieve, with the aim of quantifying their relative roles. For nonsynonymous changes, our results are consistent with both adapt and revert, and store and retrieve, contributing to the mismatch in evolutionary rates. For synonymous changes, on other hand, our results are consistent with store and retrieve contributing to the mismatch in evolutionary rates, but with adapt and revert contributing little (p24) if at all (gp41). We conclude that both mechanisms are likely to have important roles, but that these differ for synonymous and nonsynonymous substitutions, and, given the differences seen between p24 and gp41 (Figs 3 and 4), our data suggests their relative contributions also differ across genome.

Here, we have focused on the mismatch in evolutionary rates when within individual and population level rates are compared, with both rates measured across short timescales of years to a few decades. It is now well recognized that rates of viral evolution at the population level also decline as the timescales over which they are measured increase from decades to millennia [32,33]. This is often attributed to a combination of purifying selection, with the appearance and persistence of slightly deleterious mutations (independent of host genotype) over short time scales, but their eventual purging over longer time scales; and saturation effects, which are expected to be pronounced in RNA viruses due to their short genomes and high mutation rates [32–36]. Here, we also detected patterns of evolution within individuals that are consistent with purifying selection, specifically the purging of transmitted slightly deleterious mutations, which may contribute to the slowing of measured evolutionary rates at the population level as progressively longer timescales are considered. It is also possible that adapt and revert, and store and retrieve, might provide additional mechanisms leading to this slowing of measured evolutionary rates [7], but further work is needed to assess their likely importance.

A unique feature of our analysis is the number of individuals included. This makes our overall analysis more robust than those based on fewer individuals, and also highlights the heterogeneity in patterns observed among individuals as well as between the two gene regions we looked at. There is ongoing interest in trying to estimate the number of variants initiating HIV infections [15,48–53]. Our analysis highlights that focusing on a single gene region can potentially be misleading. For example, diversity measurements for individual i24 indicate infection by a single variant when looking at the p24 gene region, but multiple variants when looking at the gp41 region. The most likely scenario is that the donor individual (or i24 before the first sampling timepoint) was superinfected by a distinct variant from an unknown individual, followed by recombination in either the donor or i24, which led to the maintenance of two distinct lineages in gp41 but not p24. Similarly, there is interest in estimating time since infection from measures of within-host viral diversity [1,3,54–57]. However, diversity is not always a good measure, as it can be elevated for substantial periods of time due to the persistence of multiple founder lineages, as seen in individual i24, and can drop dramatically as a consequence of within-host population dynamics, as likely seen in individuals i24 and i17, although amplification biases cannot be ruled out.

The continual adaptation of HIV-1 to different host environments and selection at the point of transmission are both likely to contribute to the complex patterns of HIV-1 evolution observed at the within-individual and population levels. Moreover, different selection pressures acting across the genome coupled with high rates of recombination further complicate the picture [26]. In particular, recombination is likely to elevate within-host evolutionary rates as a result of generating more diverse viral lineages. Disentangling the evolutionary pressures faced by chronic viruses will not only help us to understand how selection acts across multiple ecological scales [27], but will also have direct clinical importance by shaping our understanding of pathogenesis [58], how drug- and immune-escape variants are likely to spread through populations [41,59,60], and in the development of preventive vaccines [61]. Deeply sequenced, whole-genome reads that avoid PCR generated recombination and amplification errors will be needed to fully delineate the relative roles of all of these pressures across the genome.

## Methods

### Study population

HIV-1 seroconverters participating in the Rakai Community Cohort study who were co-enrolled in the Molecular Epidemiology Research (MER) seroconverter study were previously screened for HIV-1 superinfection [37,62]. HIV-1 seroconversion date was estimated as the midpoint between the last seronegative and the first seropositive sample as tested in the Rakai Community Cohort study. Apart from i11, the first sequenced sample corresponds to the first positive sample (for i11, the first positive sample was 46 days earlier than the first sequenced sample). Individuals who had both gp41 and p24 regions sequenced for both time points previously screened, were not superinfected, and who had at least three additional serum samples available as part of the MER study were included in the study (Table 1).

### Sequencing

Previously generated sequences were used for this analysis [37]. In addition, serum samples from the three additional study time points were analyzed using identical next generation sequencing (NGS) methods, as described previously [37,63]. Briefly, HIV-1 RNA was extracted from 140μL plasma, reverse-transcribed, and amplified using a nested-polymerase chain reaction (PCR) to produce amplicons corresponding to portions of the viral p24 (~390 bp) and gp41 (~324 bp) gene regions. The corresponding HXB2 reference genome positions for p24 and gp41 used in this study are 1429–1816 and 7941–8264, respectively. Successfully amplified samples for both study visits (baseline and follow-up) in at least one region were sequenced using the 454 DNA Sequencing platform as previously described, with adjustments to use a 2-region format (Roche, Branford, CT) [37,62,63]. Pools of samples were processed using emPCR Amplification Manual-Lib-L-LV–June 2013 (Roche Branford, CT) using 25% of the recommended amplification primer amount and a 0.2 copy-per-bead ratio [63].

The resulting sequencing reads were analyzed and similar sequences were combined into a single consensus sequence. The number of reads and consensus sequences for each sample in the study, plus viral load and CD4 counts where available, are shown in S2 Table. Short sequence reads (>10 bp short of the individual consensus) were removed, and consensus sequences that encompassed a cluster of at least ten individual, near-identical sequence reads were determined and used for all subsequent analyses [37,63]. In order to remove any residual contaminating sequences a representative sequence from all distinct viral populations for each sample run in a given NGS sequencing plate were combined in a neighbor-joining tree, and any micro-contamination or spill-over sequences that localized with another unrelated sample were removed. A final manual alignment of the sequences was performed to ensure the sequences aligned within and across the different individuals for all time points, and gaps were inserted where necessary to keep the reads in-frame. One or two base-pair insertions associated with homopolymeric tracks were removed. This realignment typically reduced the number of distinct consensus sequences at each time point because the position of gaps in the sequences was standardized. Through this procedure, most errors associated with 454 sequencing of HIV were corrected for, particularly indels associated with homopolymeric regions [64]. To reduce the impact of substitution errors introduced through 454 sequencing, we excluded in our evolutionary analyses sites within individuals where the second-most frequent allele frequency was <0.056% (the estimated error-frequency per nucleotide [64]), under the assumption that polymorphisms at these sites were due to sequencing error. We also checked the resulting alignments for recombination using RDP4 [65], which did not detect any recombination breakpoints.

### Determining consensus sequence(s) at the first sampling time point

Estimating the founder strain(s) that initiated each infection is challenging because the first sampling time point for each of the individuals in our study is estimated to be between 150 and 425 days since seroconversion. A common approach is to use the consensus sequence at the first sampled time point as a proxy for the founder strain. However, if an individual was infected by multiple strains, this can give misleading estimates and add considerable noise to the data. To help remediate this effect, we identified genetically distinct subgroups at the first sampling time point using hierBAPS (Hierarchical Bayesian Analysis of Population Structure) [66]. The consensus sequence of each of these subpopulations was then determined, with these representing our proxies of the founder strain(s). We note that although sufficient for our analysis, this is not a good method to determine the actual number of founder strains in our data, with some consensus sequences from the same individual differing by only a single base in our analysis. For gp41, six individuals had more than one consensus sequence, and for p24, eight individuals had more than one consensus sequence.

### Divergence and diversity

Diversity and divergence over time were calculated on the full sequence data using custom-made Python scripts, which are available on github (https://github.com/katrinalythgoe/RakaiHIV). For divergence, we estimated the mean pairwise genetic difference at each time point between each viral gene sequence from that time point and the consensus sequence(s) from the first time point. In cases where multiple consensus sequences were estimated, we inferred the most closely related ancestral sequence by only considering the minimum pairwise genetic difference for each sequence against the available consensus sequences. Diversity corresponded to the mean pairwise genetic differences among the sequences sampled at a particular time point. For both diversity and divergence, we only considered sites with minor allele frequency greater than 0.056% (the estimated error-frequency per nucleotide from [64]).

### Estimates of within- and between-host evolutionary rates

We estimated the within- and between-host evolutionary rates using BEAST [67] by employing a hierarchical phylogenetic model (HPM) [68] and a renaissance counting approach [69]. For the within-host evolutionary analysis, this approach has been shown to yield more precise estimates (e.g. [70]), as it enables information about the evolutionary parameters (e.g. substitution model and molecular clock) to be explicitly shared among the different individual datasets while allowing independent evolutionary histories for each individual. Specifically, renaissance counting is a probabilistic counting method for estimating nonsynonymous and synonymous substitution rates and site-specific dN/dS ratios using codon-partitioned nucleotide substitution models. It is based on a stochastic mapping approach, which infers the changes (or counts) at each site in the alignment across the phylogeny using a continuous-time Markov chain (CTMC) model of nucleotide substitutions, and empirical Bayes modeling to avoid inflated standard errors of the number of substitution counts, namely by excluding counts that are either zero or infinity. Site-specific dN/dS ratios can be estimated by dividing the observed nonsynonymous (cN) and synonymous substitutions (cS) with expected nonsynonymous (uN) and synonymous changes (uS), e.g. dN/dS = (cN/cS)/(uN/uS). As this method is implemented in a Bayesian phylogenetic framework, estimates of nonsynonymous and synonymous substitution rates also take into account phylogenetic uncertainty. Furthermore, it compares well with methods that use codon substitution models, with the advantage that it is more computationally efficient. For our analysis, we first estimated posterior tree distributions for each individual (for both gene regions), using a codon-structured nucleotide substitution model [71], a strict molecular clock, and a constant tree prior, and applied noninformative hierarchical priors on the substitution, clock, and population parameters. These were subsequently used as empirical tree distributions for the renaissance counting analysis, where noninformative hierarchical priors were similarly employed for all evolutionary parameters. To reduce the computational burden of the within-host evolutionary analysis, we used a subsampled dataset for each individual, where 25 sequences per time-point were randomly selected for each gene region. The final dataset comprised of 8100 sequences where each gene-specific individual dataset ranged from 75 to 125 sequences. For the BEAST sensitivity analyses we used the CIPRES Science Gateway [72].

To estimate the between-host evolutionary rates, we collated independent datasets for subtypes A, C and D HIV-1 infections from Uganda using the HIV LANL database. The sequences were subsequently randomly sampled, resulting in approximately 200 sequences in each dataset. For the subtype C dataset, there were fewer sequences available from Uganda (specifically, 43 and 90 respectively for p24 and gp41 gene regions). However, these datasets were considered to have sufficient temporal structure (along with subtypes A and D), as evaluated by root-to-tip regression method in TempEst [73]. Furthermore, the viral gene sequences corresponded to the same gene regions used in the within-host sequencing study. For this analysis, we employed BEAST using a codon-structured substitution model [71], uncorrelated log-normal distributed molecular clock [74], and a Bayesian skygrid prior [75]. Nonsynonymous and synonymous substitution rates were estimated using a similar approach outlined for the within-host evolutionary analysis.

### Association between set-point viral load and within-host evolutionary rate

SPVL was calculated using similar criteria to [76], by taking the mean log_10_ viral load from all visits where viral load measurements were available, which were more than 6 months after the estimated date of seroconversion, and before the initiation of antiretroviral therapy or the onset of AIDS. This included viral load measurements taken from additional visits to those for which sequence data is available. The first viral load measurement from three individuals (i6, i15, and i20) was excluded because they were more than ten times higher than all subsequent measurements, indicating these individuals were in acute infection at the time (i.e. seroconverted soon before the first seropositive sample, rather than at the mid-point between the last seronegative and first seropositive samples, as assumed).

The mean within-host evolutionary rates among the external, internal, and backbone branches for each individual were estimated from a subset of 500 posterior trees using a custom-made script in Java (https://github.com/katrinalythgoe/RakaiHIV), which depends on the Java Evolutionary Biology Library available from https://sourceforge.net/projects/jebl/. These estimates have been summarized in S3 Table. In line with Lemey *et al*. [10], association between evolutionary rate and SPVL was examined with a Pearson correlation test at the 5% significance level.

### Evolution towards population consensus

We first calculated the population consensus sequences for subtypes A, D and C using the same sequences used to calculate the between-host evolutionary rates. For each of the 34 individuals in our study, we limited our analysis to sites that were fixed or nearly fixed for a single base at the first time point (>99% frequency). Of these sites, we defined them to be polymorphic for a given sampling time period (between 0 and 2 years since seroconversion; between 2 and 4 years since seroconversion; or over 4 years since seroconversion) if a mutation had reached an appreciable frequency (>10%) at least once during that period. For each sampling time period, we pooled data across all individuals and calculated the proportion of the changes at polymorphic sites that were towards the subtype-specific consensus for all mutations, synonymous mutations and nonsynonymous mutations (Fig 4). Additionally, we calculated the expected proportion of mutations towards the subtype-specific population consensus at polymorphic sites, assuming no selection, and a transition to transversion ratio of 2, thus accounting for a higher number of transitions in the absence of selection [77]. The bias towards population consensus was then calculated as the proportion of mutations towards subtype-specific population consensus, divided by the expected proportion of mutations towards population consensus (Fig 4). In S6 Fig, we also show the bias towards consensus for polymorphic sites assuming different transition to transversion ratios (0.5 and 4), and for all sites (not just polymorphic sites). In addition, we calculated the proportion of mutations towards population consensus at sites that were not at population consensus at the first time point (S6 Fig).

### Expected mismatch in evolutionary rates if founder-like virus is preferentially transmitted

For each sampled time point, we calculated the expected contribution to the mismatch in evolutionary rate, conditional on transmission occurring, for all, synonymous and nonsynonymous mutations. Using a similar reasoning to [11], we assumed that each sequence has a transmission fitness *w*_*d*_ = *e*^−*α d*^, where *α* determines how rapidly transmissibility declines as the distance from the consensus sequence(s) from the first sampling time point, *d*, increases. Where multiple consensus sequences were inferred, we assumed the ancestor to a given sequence was the genetically most similar one, including both synonymous and nonsynonymous mutations. The contribution to the mismatch was then calculated as the ratio of the mean number of mutations from the founder population, *μ*_*X*_, to the expected mean distance of transmitted virus from the founder population, *μT*_*X*_, giving *m*_*X*_ = *μ*_*X*_/*μT*_*X*_. Here, *X*, refers to all, synonymous, or nonsynonymous mutations. Letting *n*_*X*,*d*,*δ*_ represent the number of sequences distance *d* from the appropriate consensus sequence and that also harbor *δ* all, synonymous or nonsynonymous mutations, we can calculate *μ*_*X*_ = (∑_*d*,*δ*_ *δ n*_*X*,*d*,*δ*_)/(∑_*d*,*δ*_ *n*_*X*,*d*,*δ*_), and *μT*_*X*_ = (∑_*d*,*δ*_ *δ w*_*d*_ *n*_*X*,*d*,*δ*_)/(∑_*d*,*δ*_ *w*_*d*_ *n*_*X*,*d*,*δ*_). When calculating the mean mismatch for a given time interval, for each individual we chose the mean mismatch calculated for all the sampled time points within that interval, to avoid the frequency of sampling from biasing the results.

### Ethics statement

This project used stored samples from the Rakai Community Cohort Study in Uganda. All subjects involved in the study were adult and provided written informed consent for their samples to be stored and used for future unspecified HIV-related research. The study was approved by the Science and Ethics Committee of the Uganda Virus Research Institute, the Western Institutional Review Board, and the Committee on Human Research at the Johns Hopkins Bloomberg School of Public Health. All samples were anonymised.

## Supporting information

S1 Fig Diversity and divergence over time, with individuals infected by multiple variants removed. This is identical to Fig 1, but with individuals i1, i2, i4, i9, i12, i14, i20, i25 and i34 removed since they show high diversity in the p24 gene region at the first sampling time point, indicative of infection by multiple variants from the same donor individual. Top Row: Mean pairwise diversity at first, second, and third codon positions over time for individuals (represented in yellow, pink, and light blue, respectively). The average change in mean pairwise diversity over time was inferred by linear regression. Bottom Row: Mean nonsynonymous (purple) and synonymous divergence (blue) over time for individuals.
(PDF)

S2 Fig Patterns of diversity and divergence over time for 34 individuals. A) Mean pairwise diversity over time at first, second, and third codon positions (top, middle, and bottom panels, respectively). B) Mean nonsynonymous and synonymous divergence over time (top and bottom panels, respectively).
(PDF)

S3 Fig Time-scaled phylogenies for individual i24. Left: p24 gene tree. Right: gp41 gene tree. Numbers on the branches correspond to the posterior support (or posterior probability).
(PDF)

S4 Fig Time-scaled phylogenies for individual i17. Left: p24 gene tree. Right: gp41 gene tree. Numbers on the branches correspond to the posterior support (or posterior probability).
(PDF)

S5 Fig Mean nonsynonymous (red) and synonymous (blue) substitution rates for p24 and gp41 gene regions. The horizontal black lines correspond to overall mean for each gene region.
(PDF)

S6 Fig Comparison of within-host evolutionary rates estimated from the original analysis (red), based on 34 individuals, and from a subset of 10 individuals (blue). (PDF)

S7 Fig Comparison of within-host evolutionary rates estimated using the full codon substitution model (red) and the renaissance counting method (blue). Solid lines and filled circles correspond to the nonsynonymous substitution rates, while dashed lines and open circles correspond to the synonymous substitution rates.
(PDF)

S8 Fig Comparison of within-host evolutionary rates estimated with three different gamma hyperpriors (defined by scale parameters 10, 100, and 1000, respectively) for the clock rate hierarchical model (see main text for details). (PDF)

S9 Fig Scatter plot of mean within-host evolutionary rates and set-point viral load. For both gene regions, we estimated the mean within-host evolutionary rates for external, internal, and backbone branches at both nonsynonymous (filled circles) and synonymous (open circles) sites. The points are coloured according to subtype as per Fig 2. The solid and dashed lines indicates the best linear fit for nonsynonymous and synonymous substitution rates, respectively, and set-point viral load. Using a Pearson correlation test, we found no significant relationship between evolutionary rate and set-point viral load in this cohort.
(PDF)

S10 Fig Changes towards population consensus. For each gene region, the figures give: the proportion of polymorphic sites where a mutant allele represents a change towards the subtype-specific population consensus; the bias towards subtype-specific population consensus for polymorphic sites, with assumed mutational transition:transversion (ts:tv) ratios of 0.5, 2 and 4; the proportion of polymorphic sites that are non-consensus at the first time point, which change towards subtype-specific consensus; and the bias towards subtype-specific population consensus for all sites, with assumed mutational transition:transversion ratios of 0.5, 2 and 4. In all cases, the error bars give the 5 and 95 percentiles from 10,000 bootstraps of the individual data. Black, all changes; Blue, synonymous changes; Red, nonsynonymous changes.
(PDF)

S11 Fig Changes towards population consensus, with individuals probably infected by multiple variants removed. This is identical to S10 Fig, but with individuals i1, i2, i4, i9, i12, i14, i20, i25 and i34 removed since they show high diversity in the p24 gene region at the first sampling time point, indicative of infection by multiple variants from the same donor individual. In addition, i24 was also removed, due to very high diversity in gp41. In all cases, the error bars give the 5 and 95 percentiles from 10,000 bootstraps of the individual data. Black, all changes; Blue, synonymous changes; Red, nonsynonymous changes.
(PDF)

S12 Fig Contribution to the mismatch in evolutionary rates if founder-like virus has a transmission advantage, with individuals probably infected by multiple variants removed. This is identical to Fig 4, but with individuals i1, i2, i4, i9, i12, i14, i20, i25 and i34 removed since they show high diversity in the p24 gene region at the first sampling time point, indicative of infection by multiple variants from the same donor individual. In addition, i24 was also removed, due to very high diversity in gp41. The error bars give the 5th and 95th percentiles from bootstrapping over the individuals 100,000 times. Black, all mutations; Blue, only synonymous mutations are considered when calculating the expected mismatch; Red, only nonsynonymous mutations are considered when calculating the expected mismatch (see Methods).
(PDF)

S1 Table Average within- and between-host rates per subtype. (DOCX)

S2 Table Viral load, CD4+ counts, and number of sequence reads per time point for all individuals. (CSV)

S3 Table Within-host evolutionary rates per individual along the backbone, internal, and external branches. (CSV)

S1 Text Full description of the BEAST sensitivity analyses. (DOCX)
