# Supplementary figures and images for: Evolution of HIV-1 within untreated individuals and at the population scale in Uganda
Source: PLoS Pathog. 2018 Jul 27;14(7):e1007167. doi: 10.1371/journal.ppat.1007167 (PMC6082572; doi:10.1371/journal.ppat.1007167)

p24

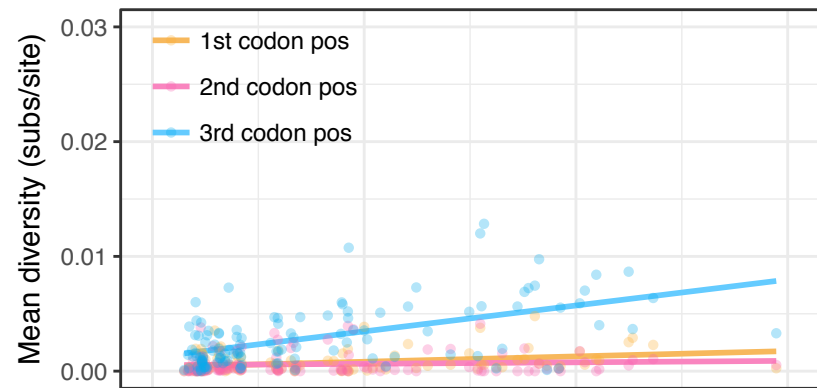

gp41

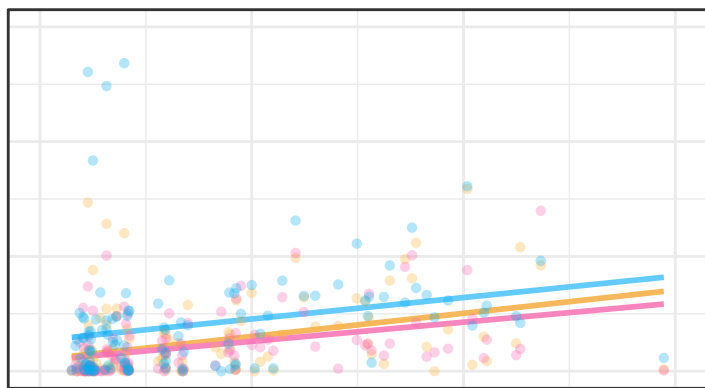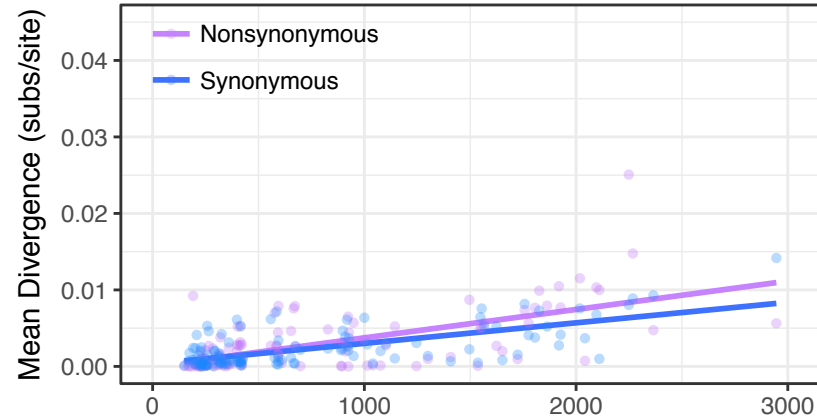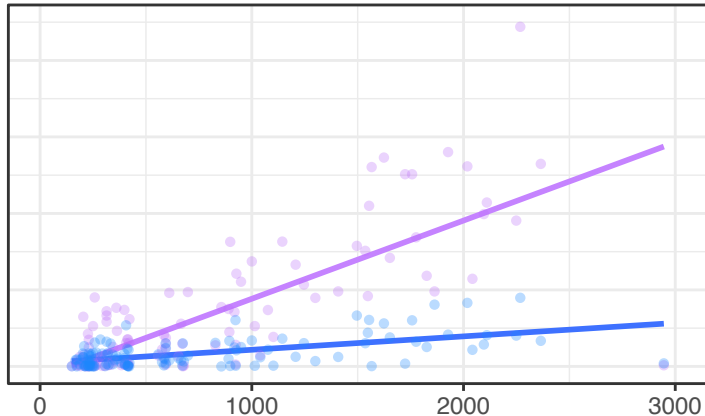

Time (days post seroconversion)

Supplement: S1 Fig — This is identical to Fig 1, but with individuals i1, i2, i4, i9, i12, i14, i20, i25 and i34 removed since they show high diversity in the p24 gene region at the first sampling time point, indicative of infection by multiple variants from the same donor individual. Top Row: Mean pairwise diversity at first, second, and third codon positions over time for individuals (represented in yellow, pink, and light blue, respectively). The average change in mean pairwise diversity over time was inferred by linear regression. Bottom Row: Mean nonsynonymous (purple) and synonymous divergence (blue) over time for individuals. (PDF) [file ppat.1007167.s001.pdf]

**A**

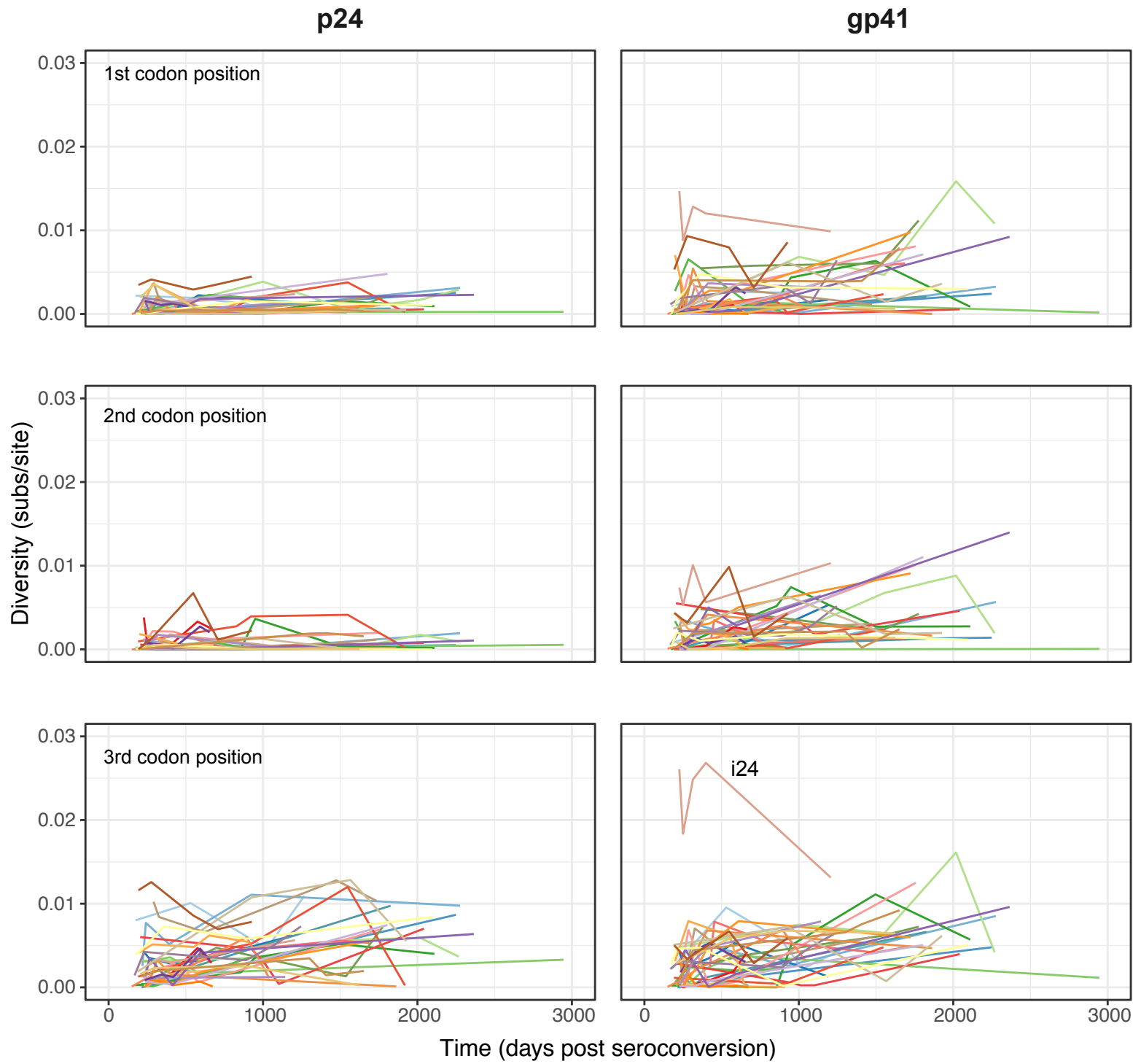

**B**

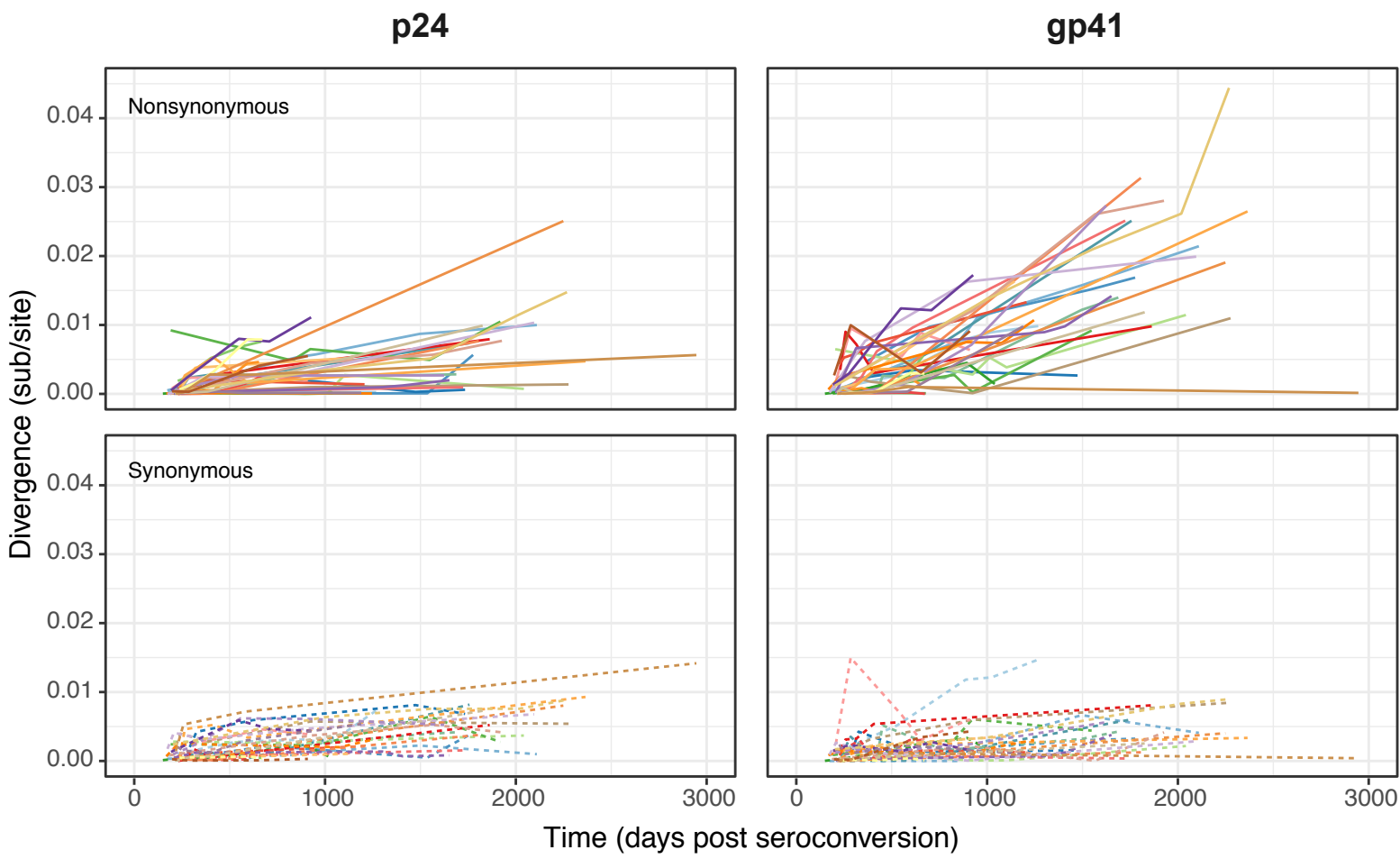

Supplement: S2 Fig — A) Mean pairwise diversity over time at first, second, and third codon positions (top, middle, and bottom panels, respectively). B) Mean nonsynonymous and synonymous divergence over time (top and bottom panels, respectively). (PDF) [file ppat.1007167.s002.pdf]

**p24**

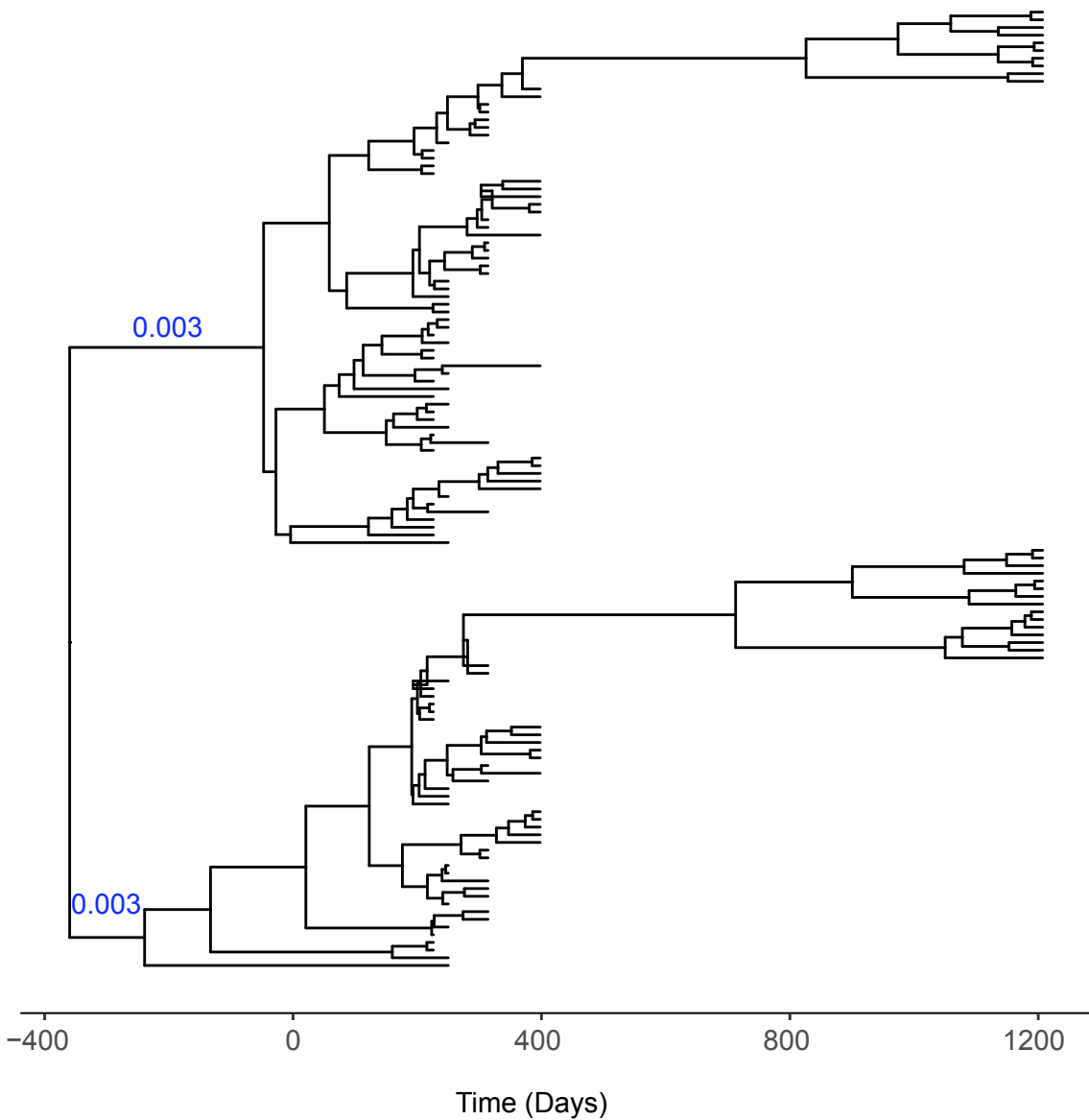

**gp41**

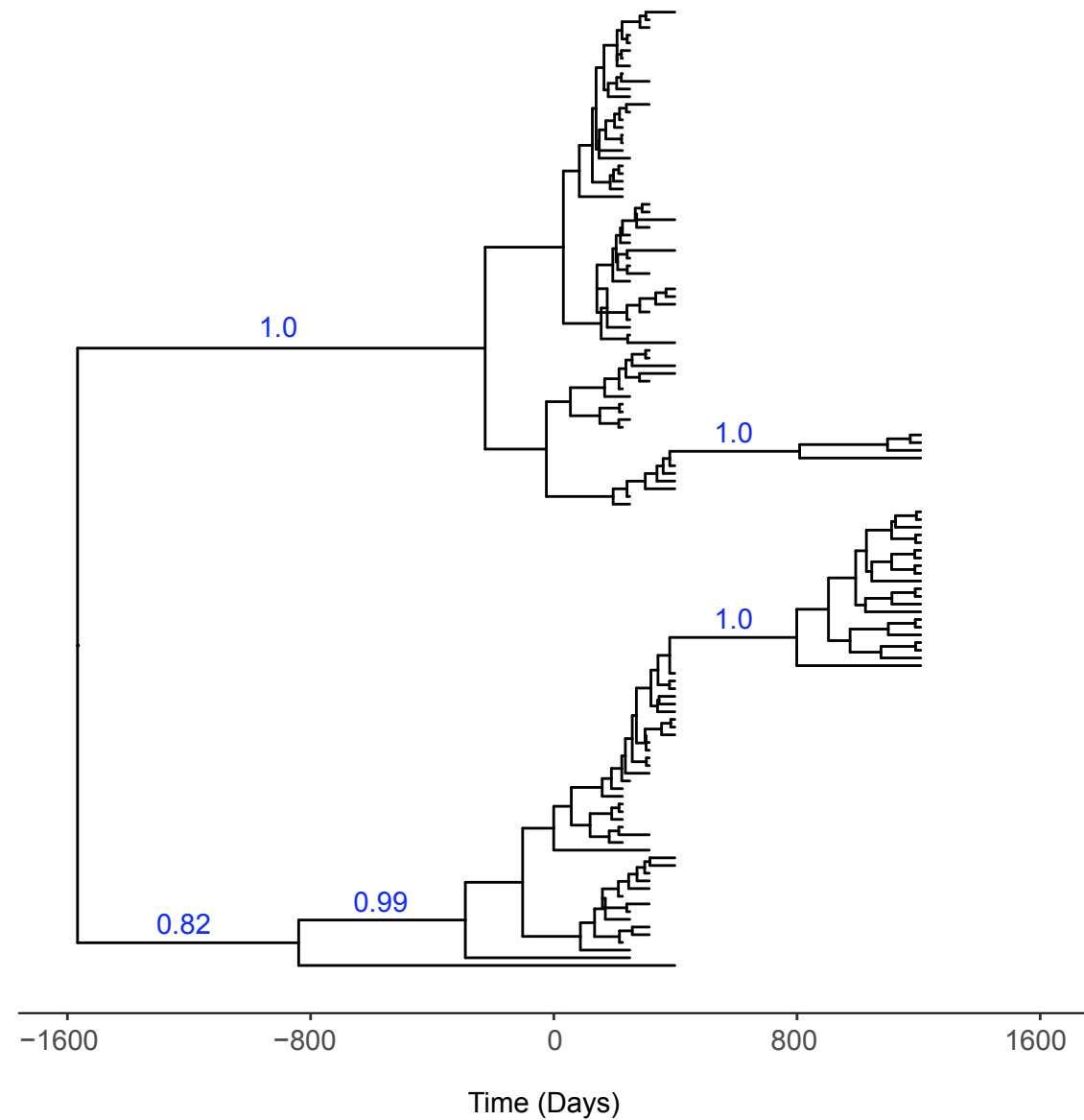

Supplement: S3 Fig — Left: p24 gene tree. Right: gp41 gene tree. Numbers on the branches correspond to the posterior support (or posterior probability). (PDF) [file ppat.1007167.s003.pdf]

**p24**

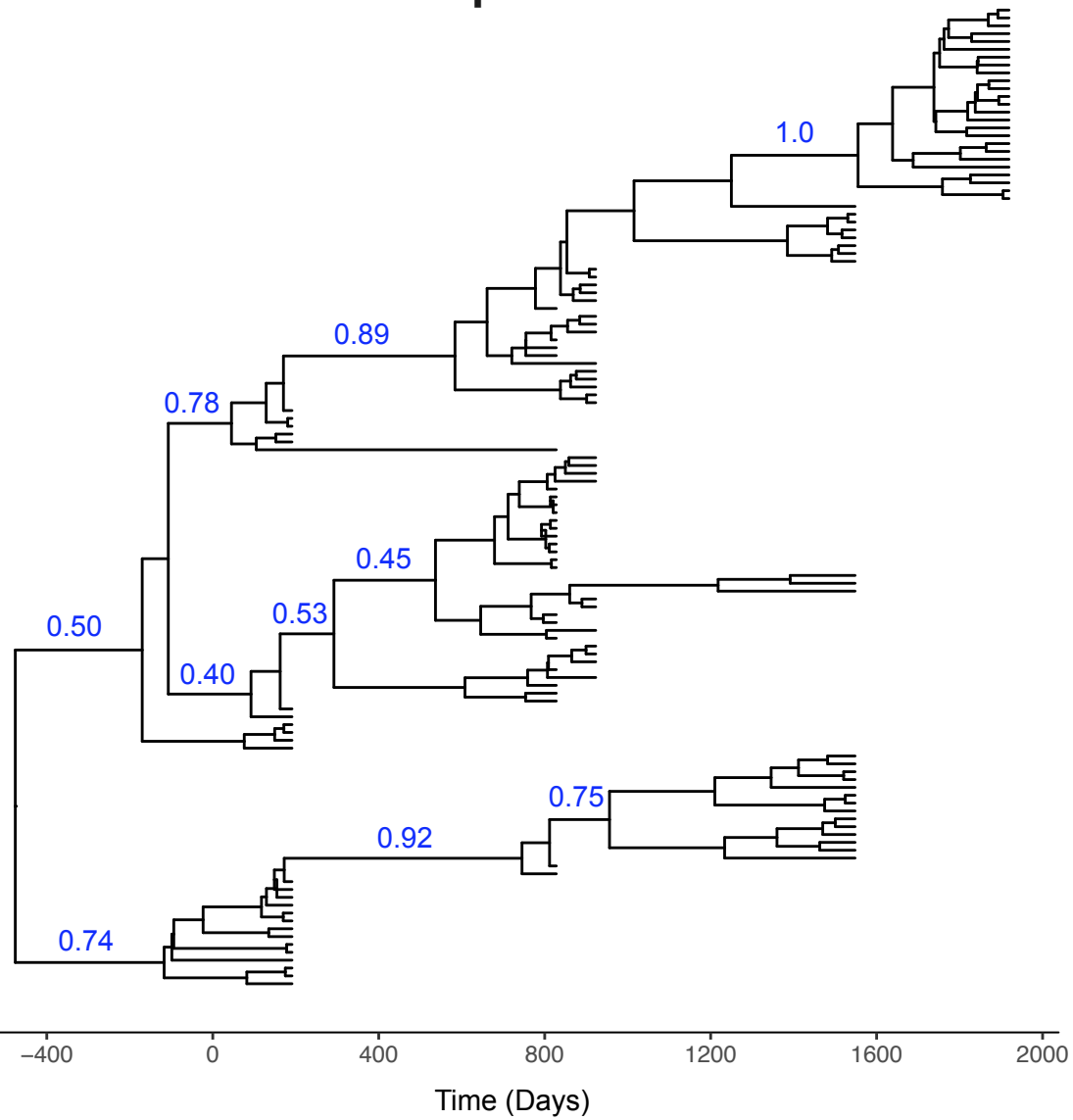

**gp41**

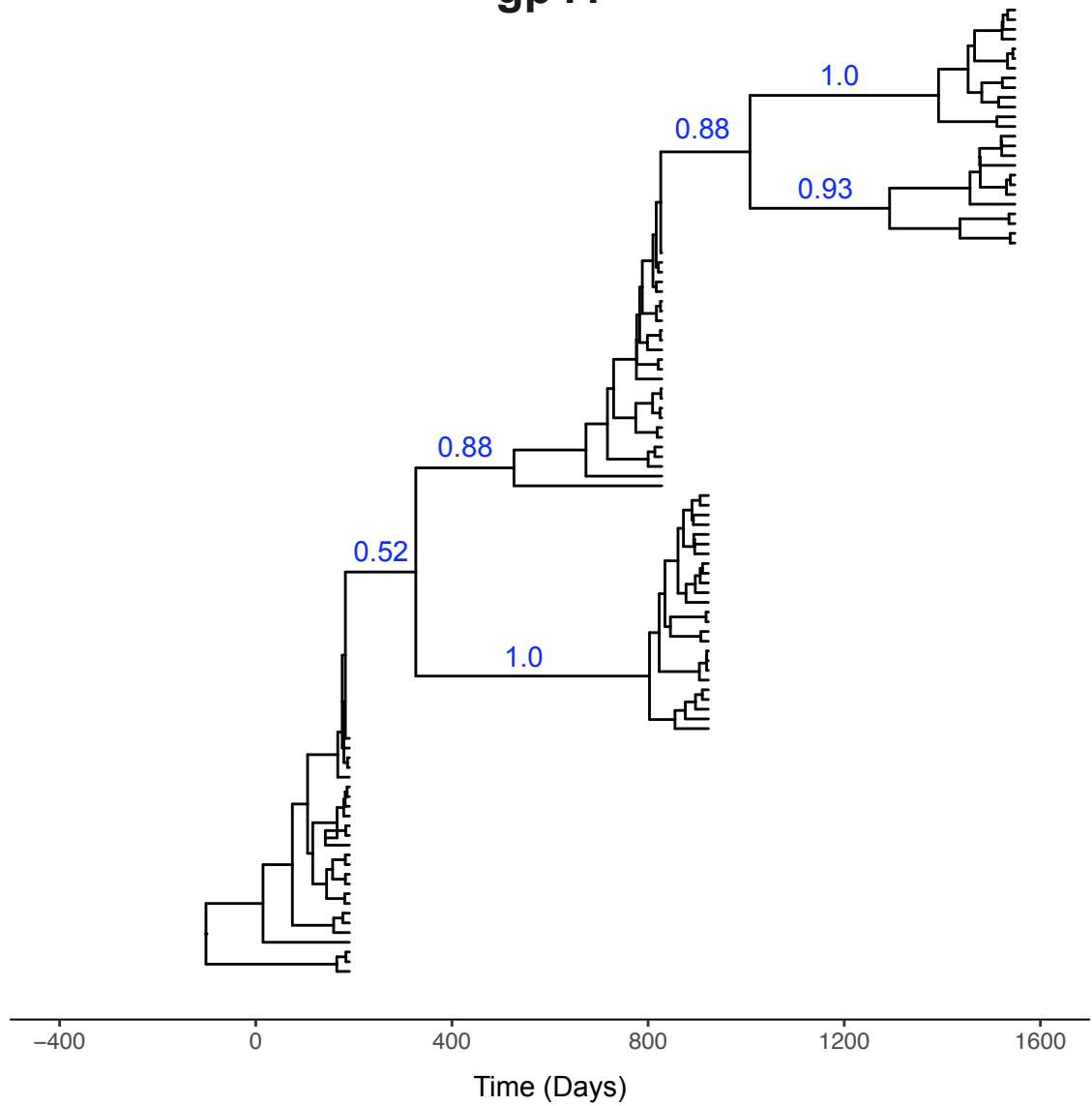

Supplement: S4 Fig — Left: p24 gene tree. Right: gp41 gene tree. Numbers on the branches correspond to the posterior support (or posterior probability). (PDF) [file ppat.1007167.s004.pdf]

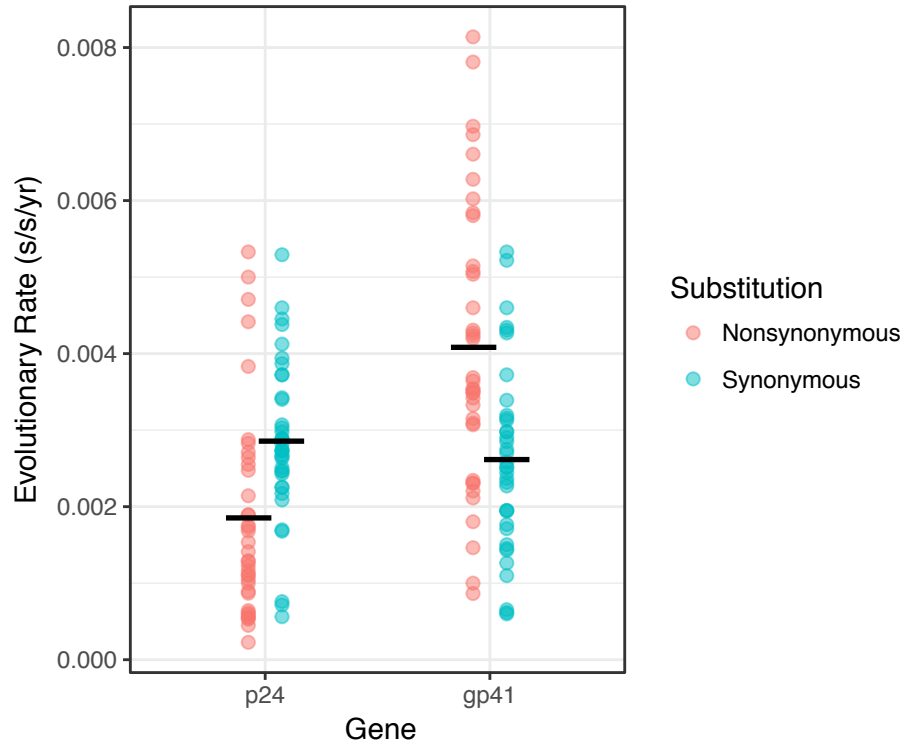

Supplement: S5 Fig — The horizontal black lines correspond to overall mean for each gene region. (PDF) [file ppat.1007167.s005.pdf]

p24

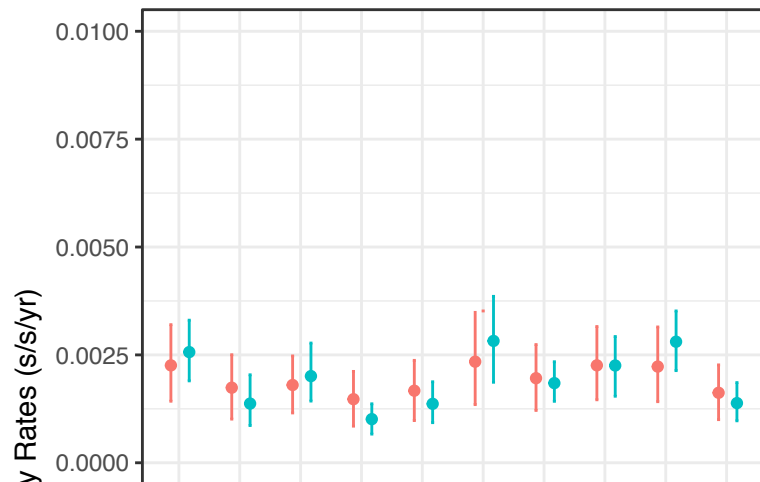

gp41

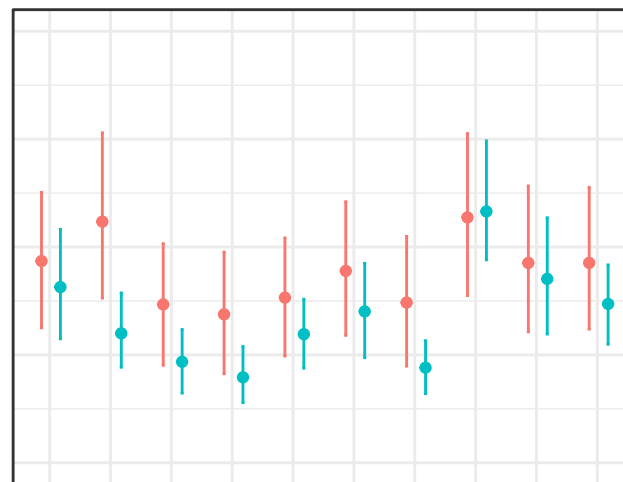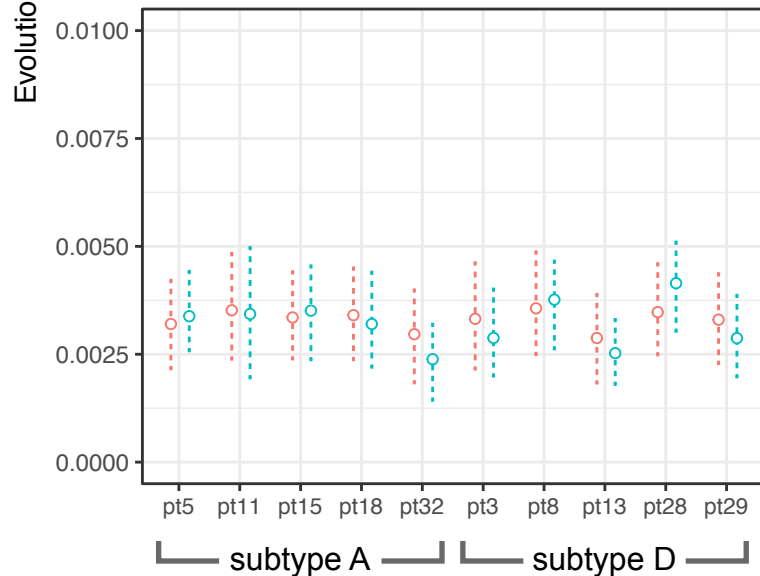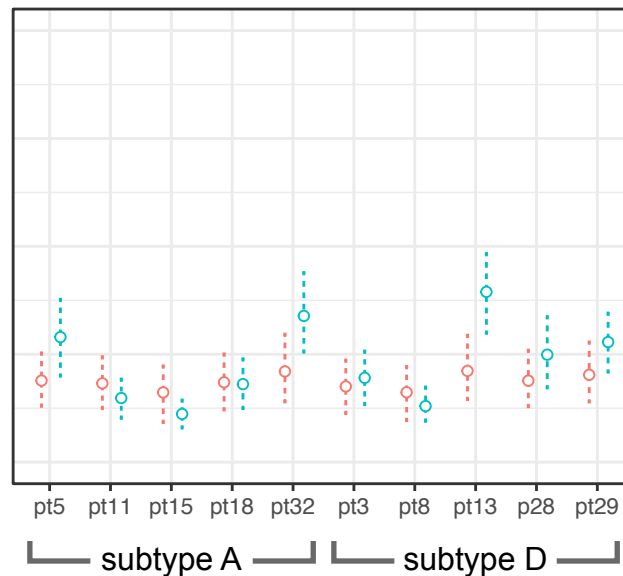

Supplement: S7 Fig — Solid lines and filled circles correspond to the nonsynonymous substitution rates, while dashed lines and open circles correspond to the synonymous substitution rates. (PDF) [file ppat.1007167.s007.pdf]

p24

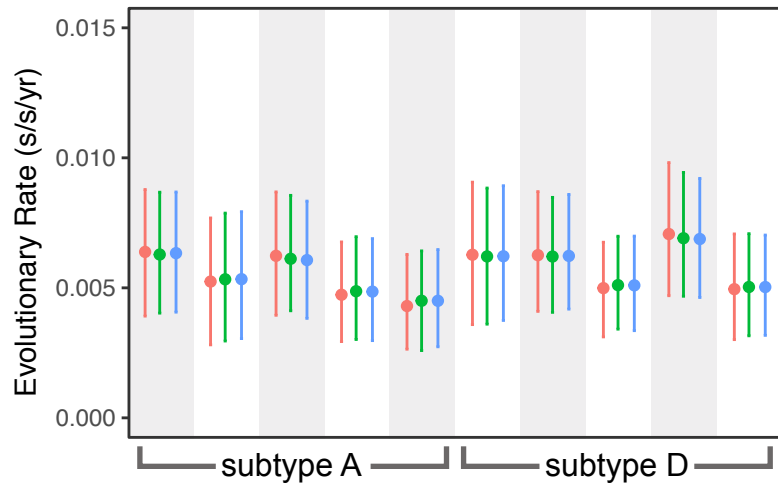

gp41

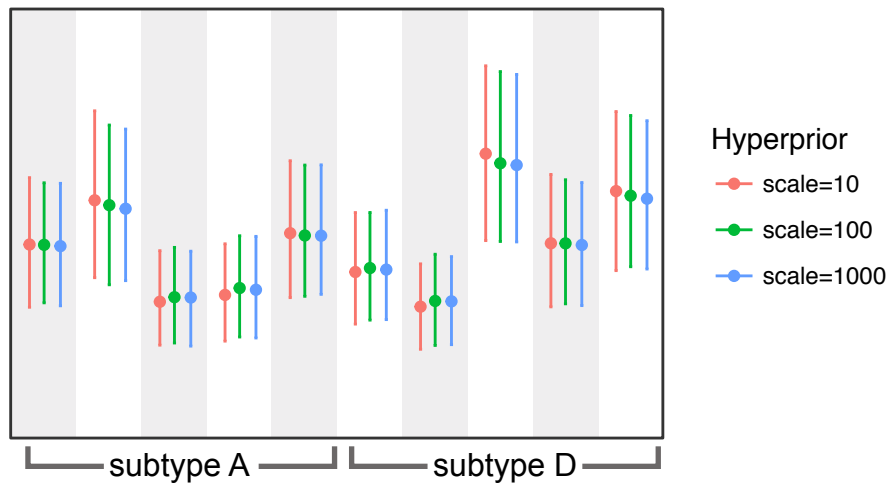

Supplement: S8 Fig — (PDF) [file ppat.1007167.s008.pdf]

**p24**

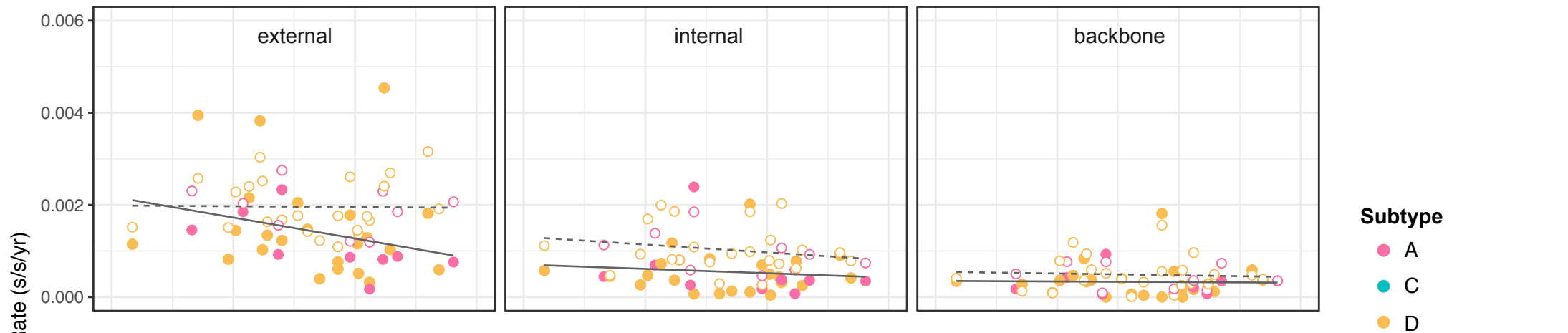

**gp41**

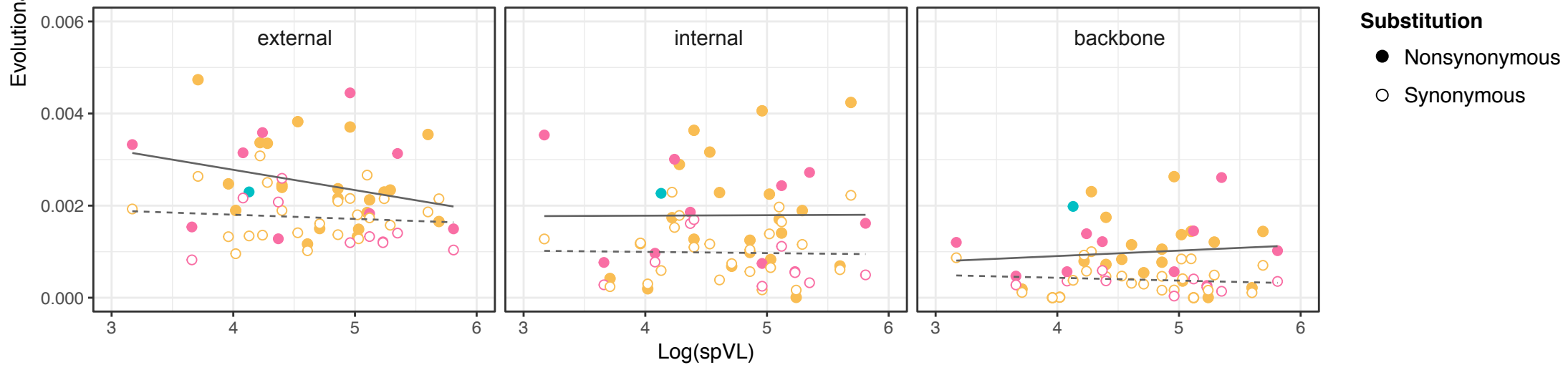

Supplement: S9 Fig — For both gene regions, we estimated the mean within-host evolutionary rates for external, internal, and backbone branches at both nonsynonymous (filled circles) and synonymous (open circles) sites. The points are coloured according to subtype as per Fig 2. The solid and dashed lines indicates the best linear fit for nonsynonymous and synonymous substitution rates, respectively, and set-point viral load. Using a Pearson correlation test, we found no significant relationship between evolutionary rate and set-point viral load in this cohort. (PDF) [file ppat.1007167.s009.pdf]

**p24**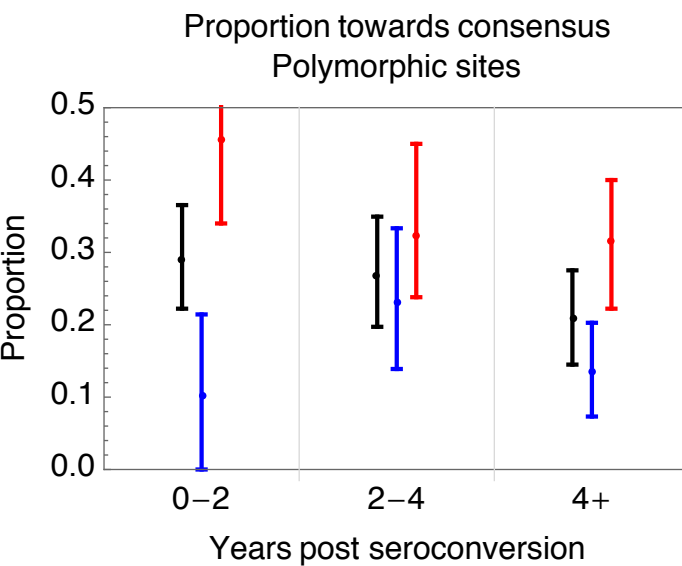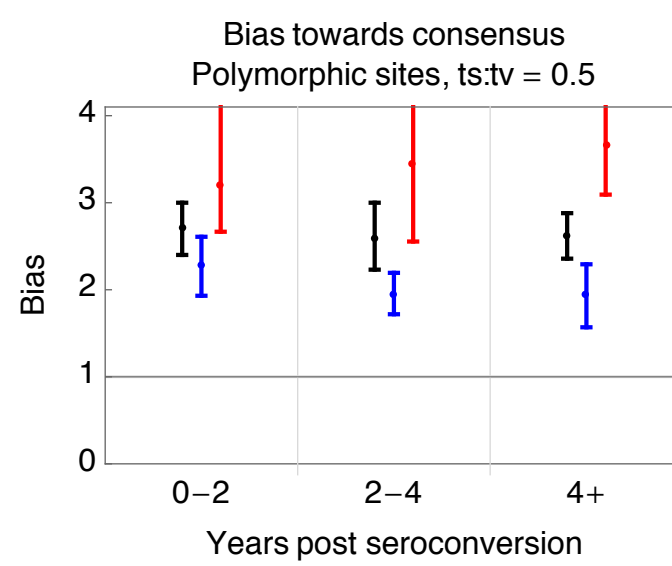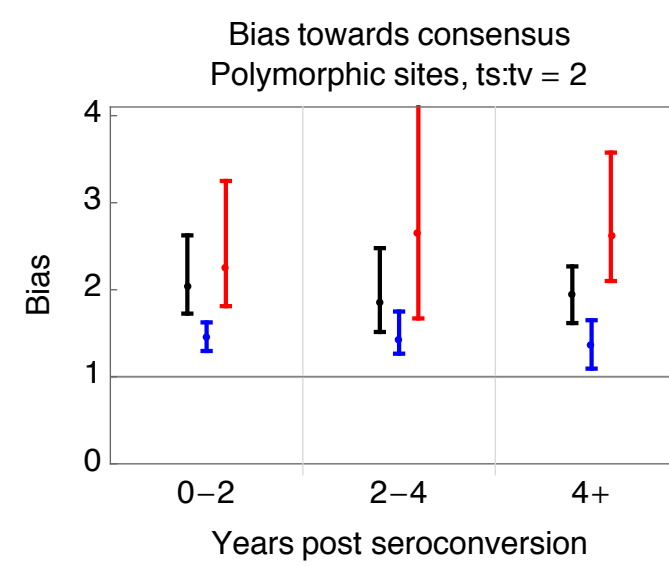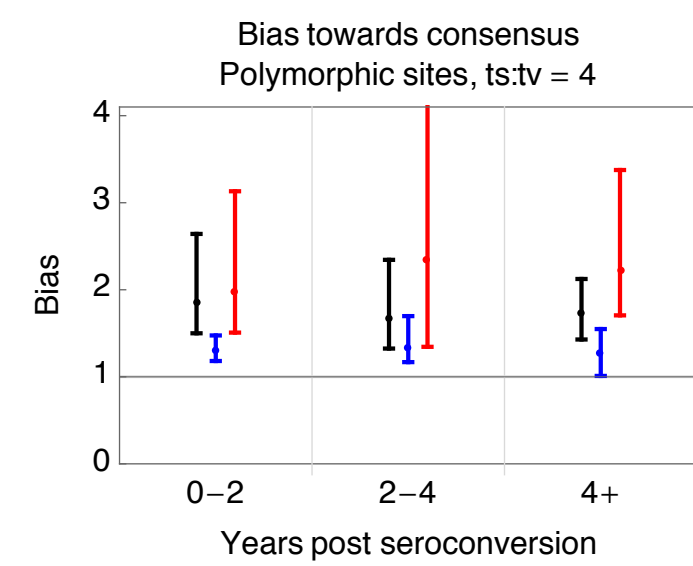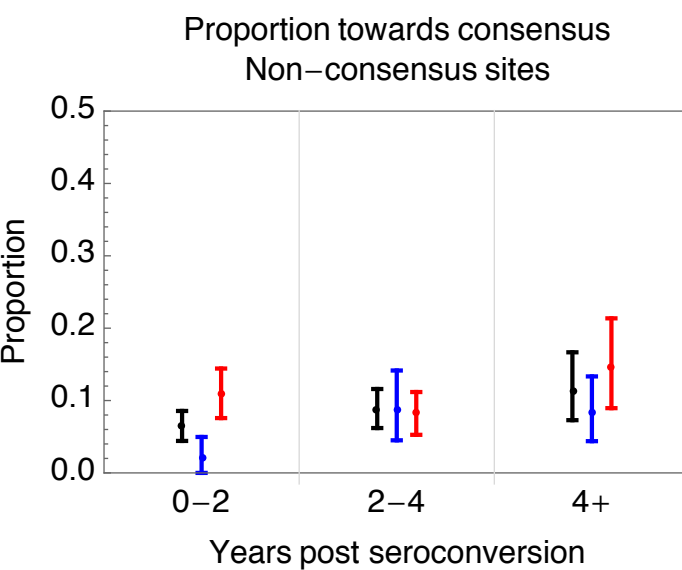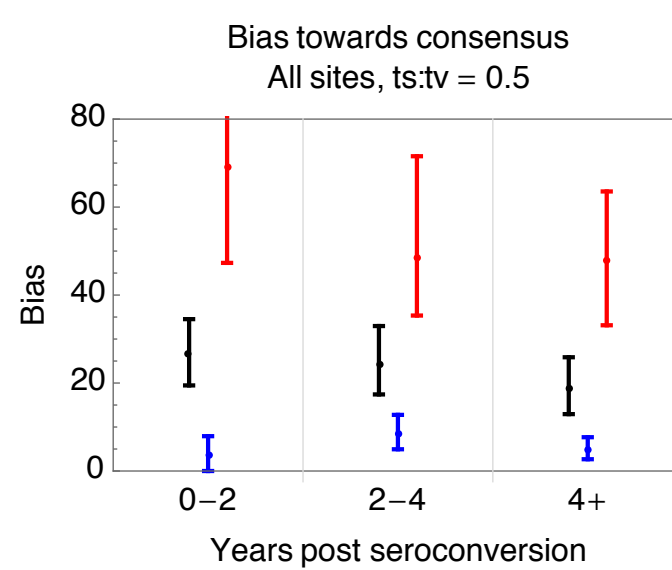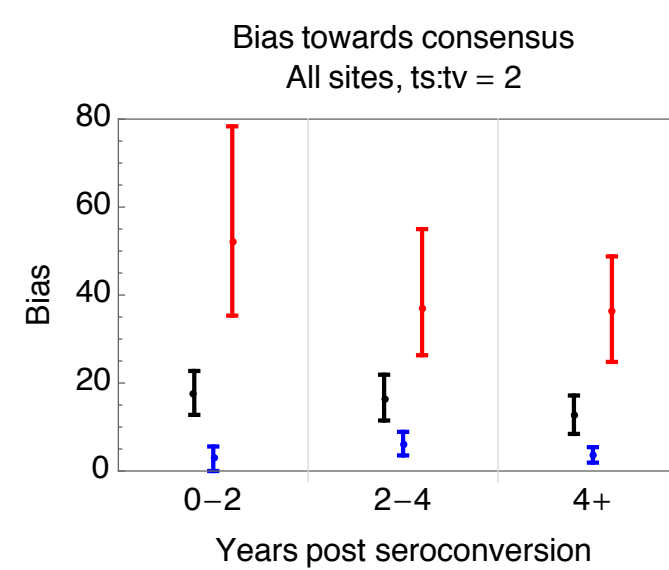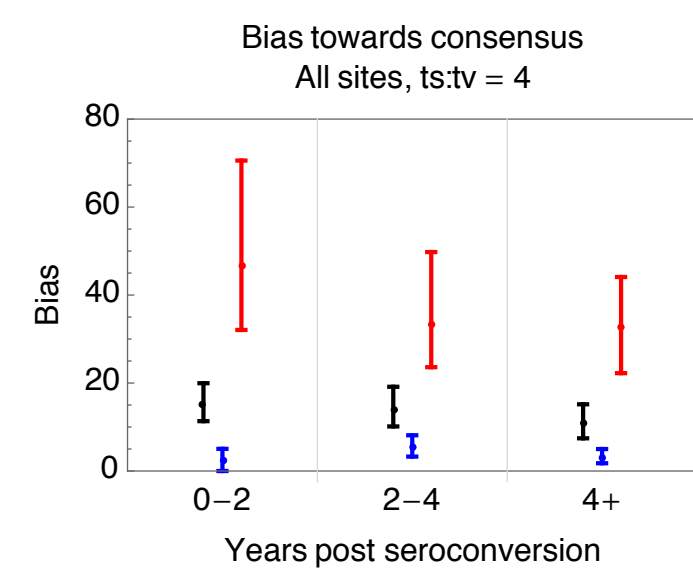**gp41**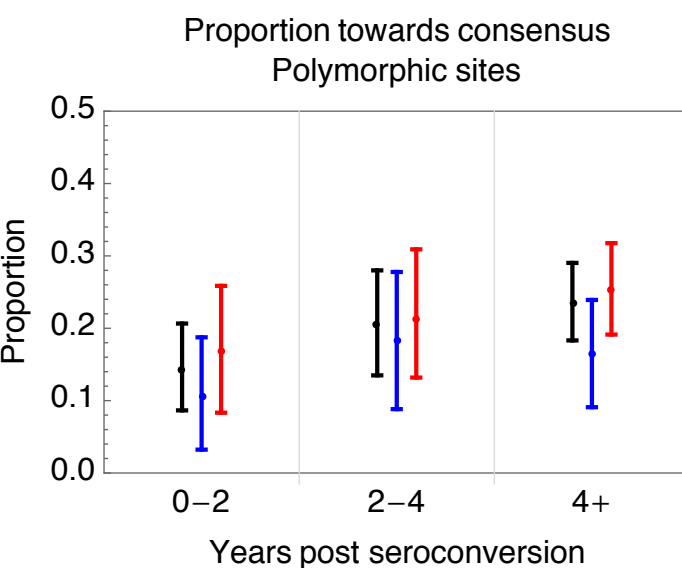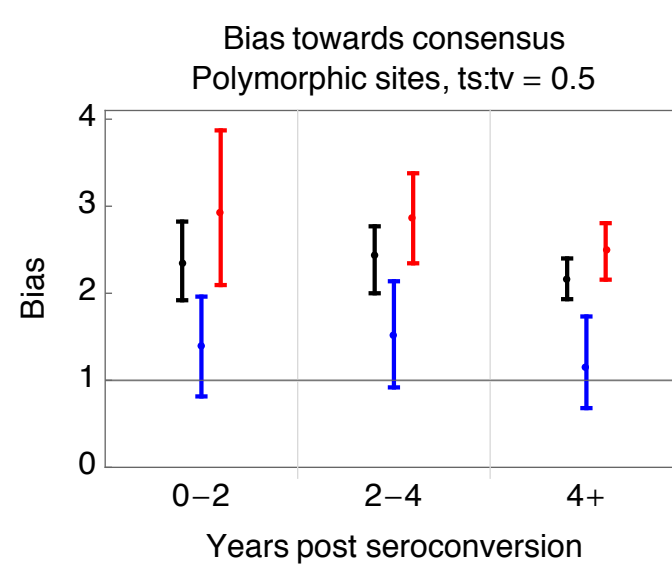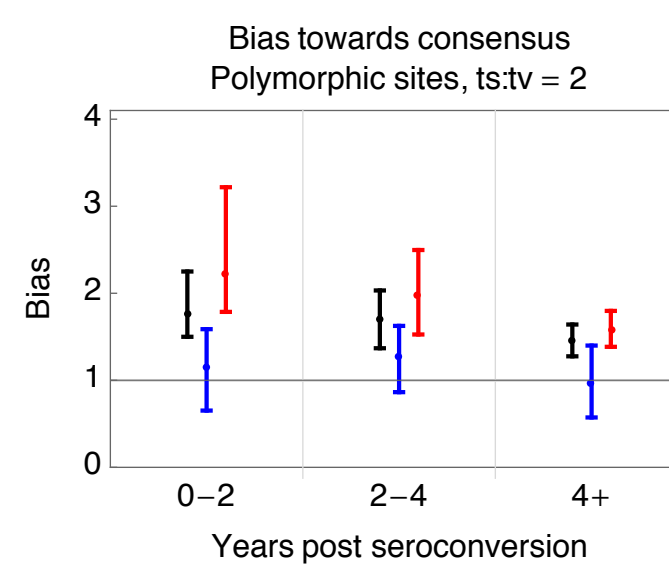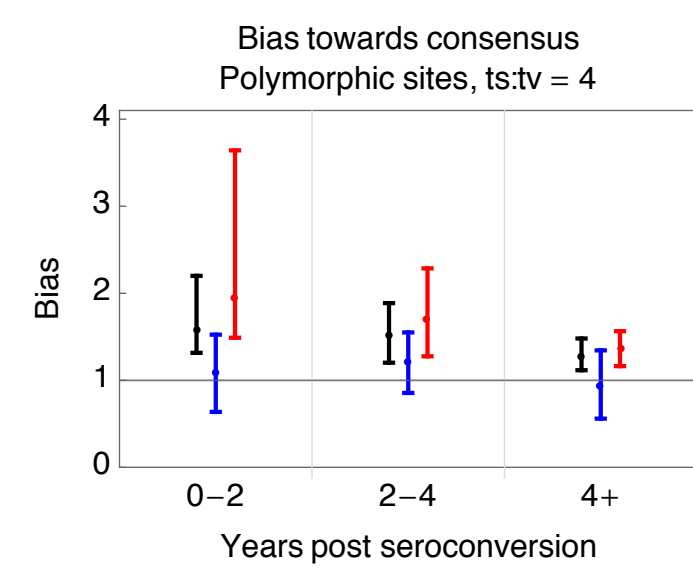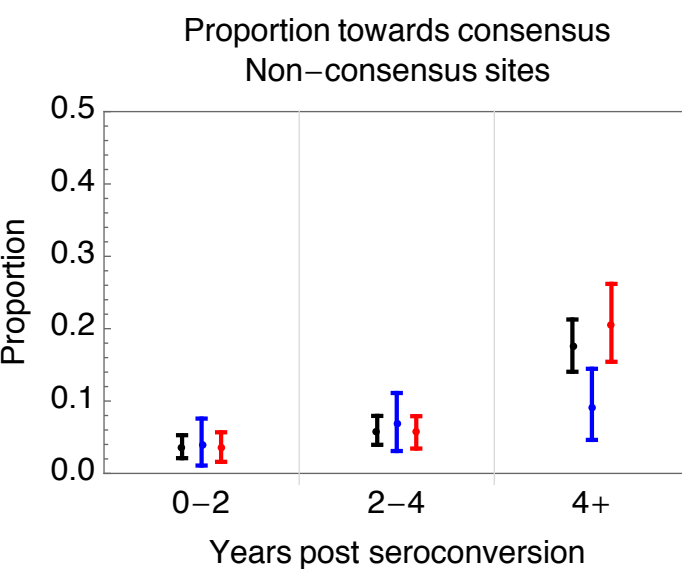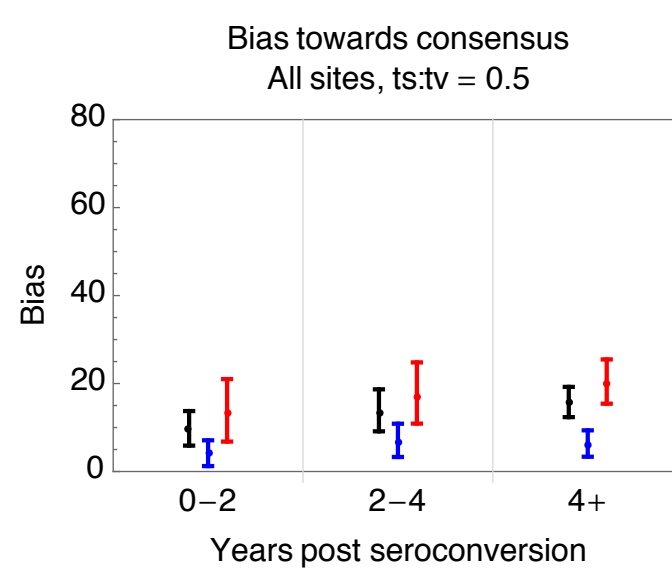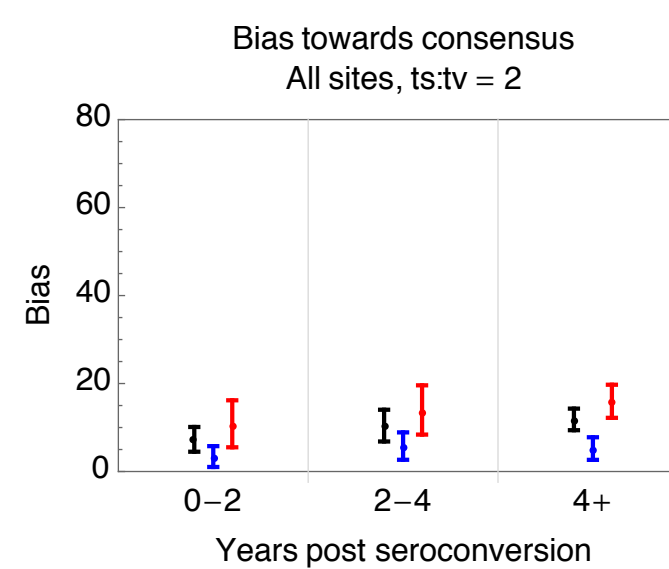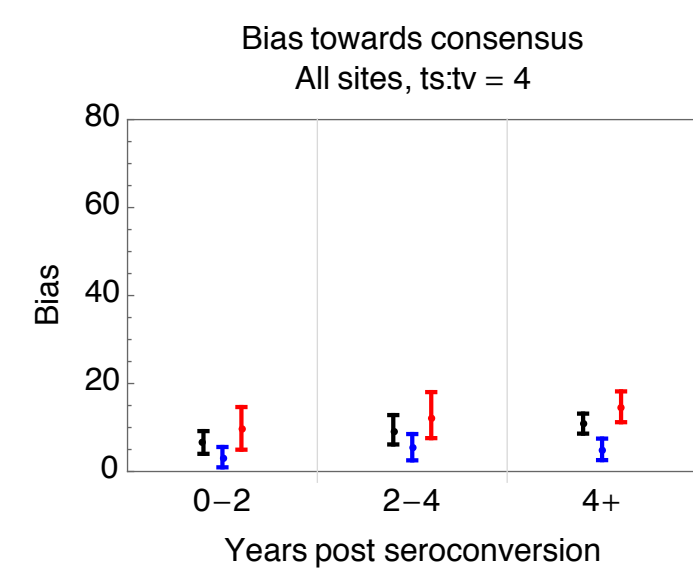

Supplement: S10 Fig — For each gene region, the figures give: the proportion of polymorphic sites where a mutant allele represents a change towards the subtype-specific population consensus; the bias towards subtype-specific population consensus for polymorphic sites, with assumed mutational transition:transversion (ts:tv) ratios of 0.5, 2 and 4; the proportion of polymorphic sites that are non-consensus at the first time point, which change towards subtype-specific consensus; and the bias towards subtype-specific population consensus for all sites, with assumed mutational transition:transversion ratios of 0.5, 2 and 4. In all cases, the error bars give the 5 and 95 percentiles from 10,000 bootstraps of the individual data. Black, all changes; Blue, synonymous changes; Red, nonsynonymous changes. (PDF) [file ppat.1007167.s010.pdf]

**p24**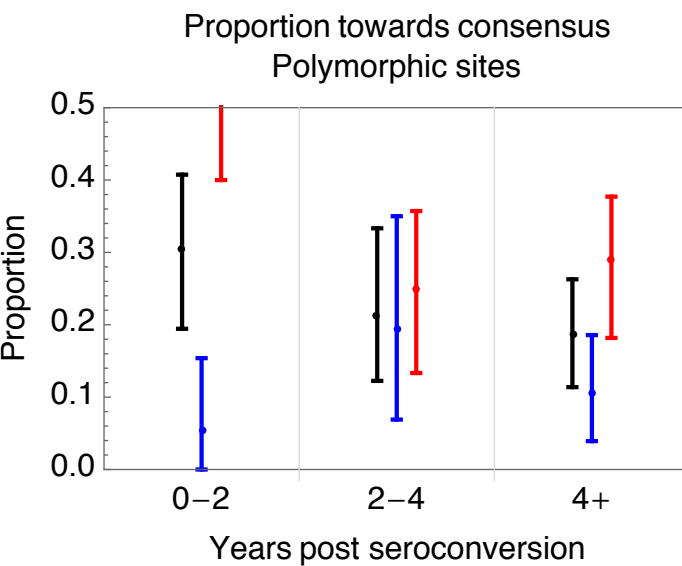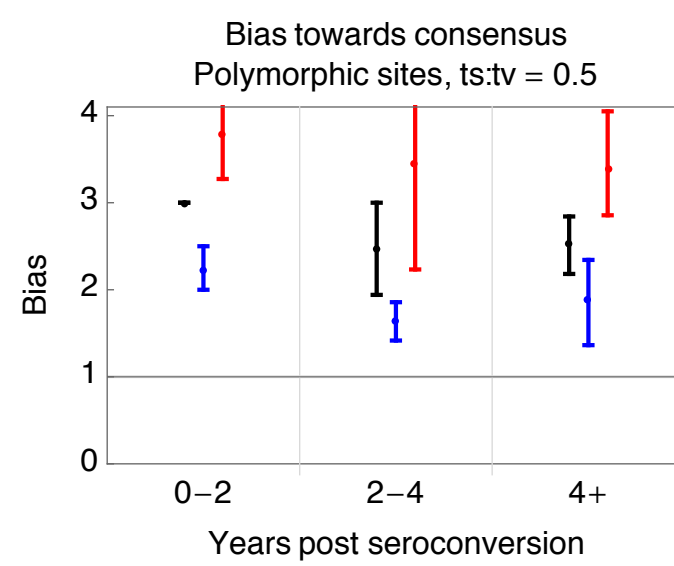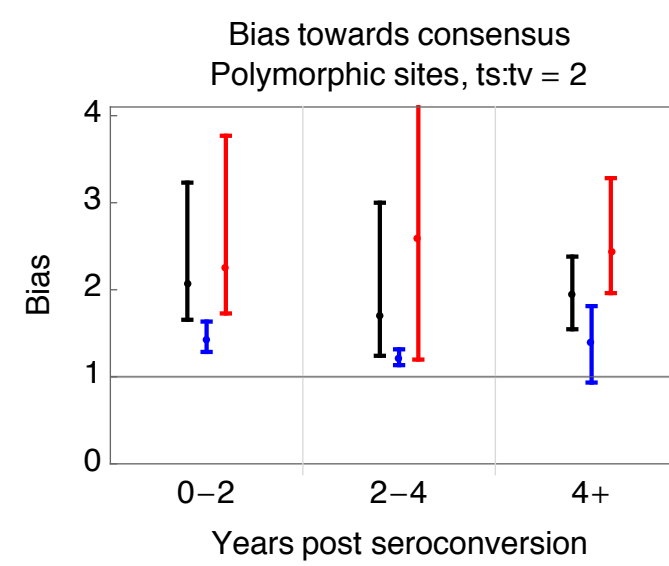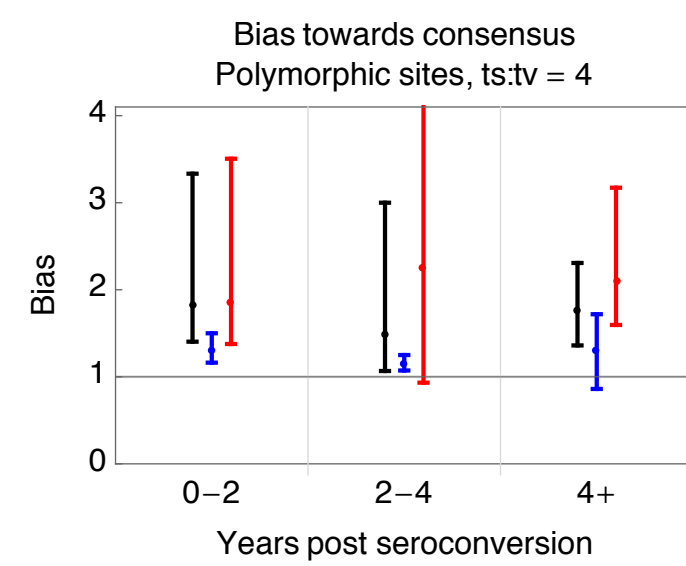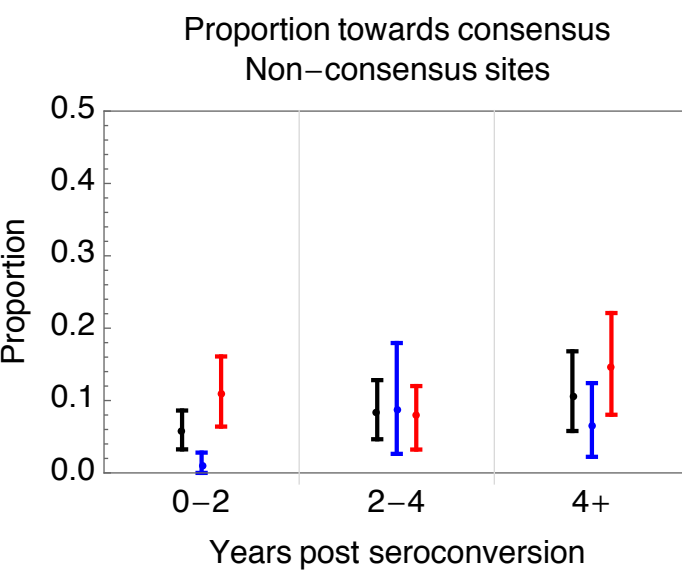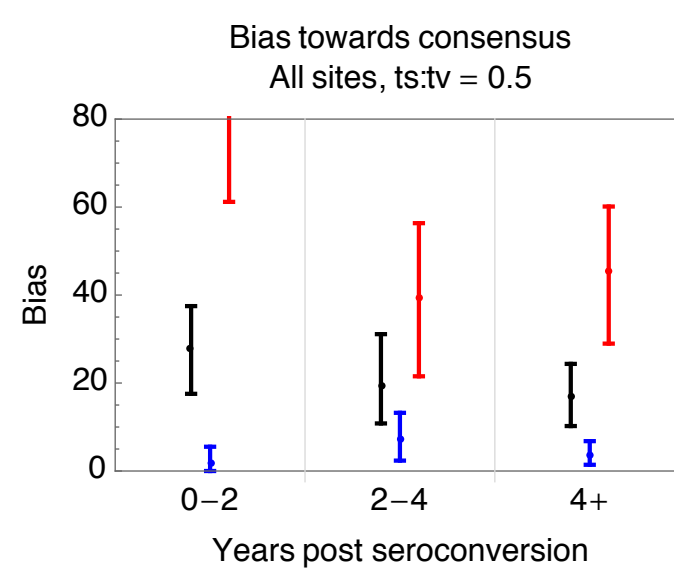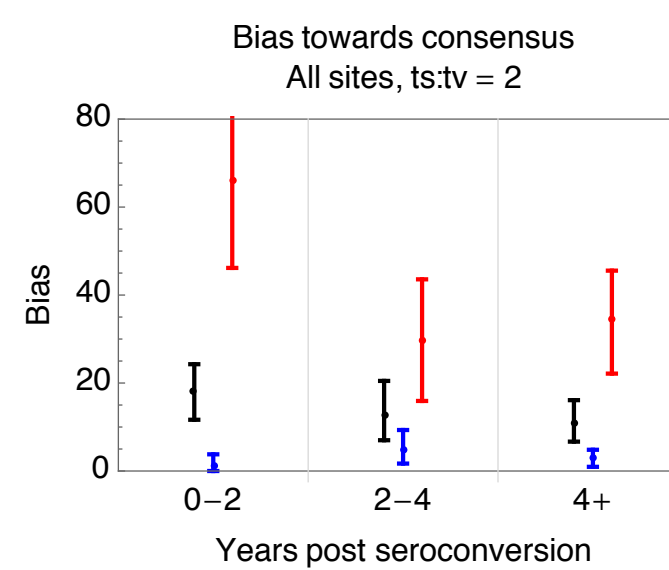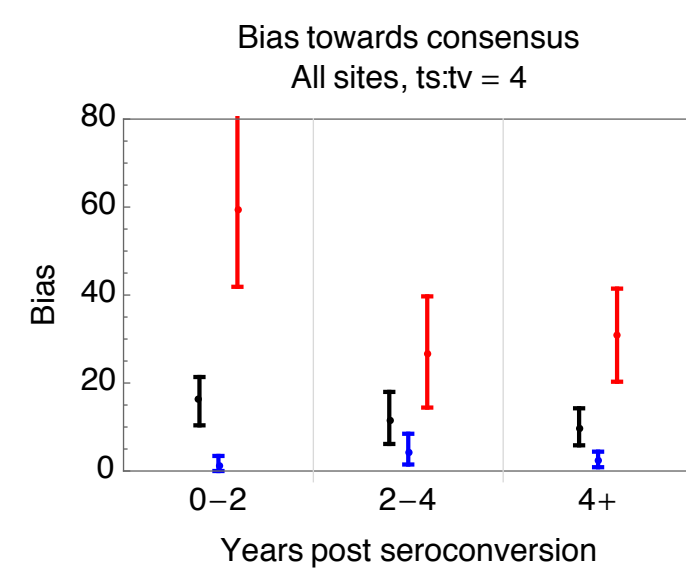**gp41**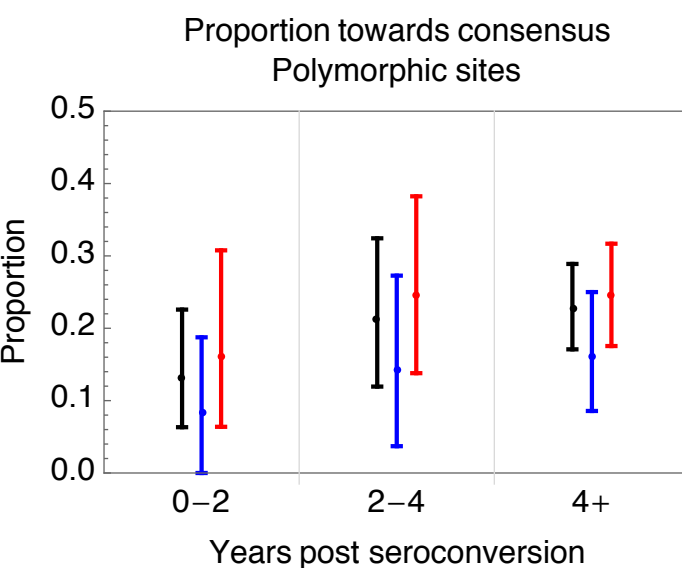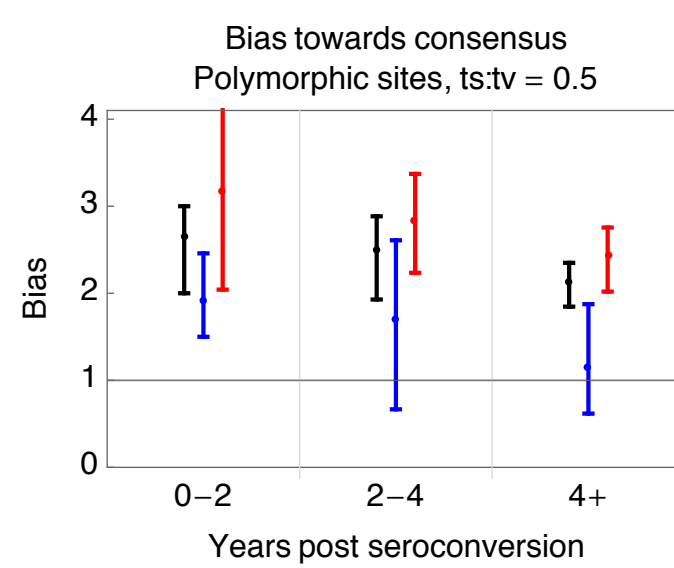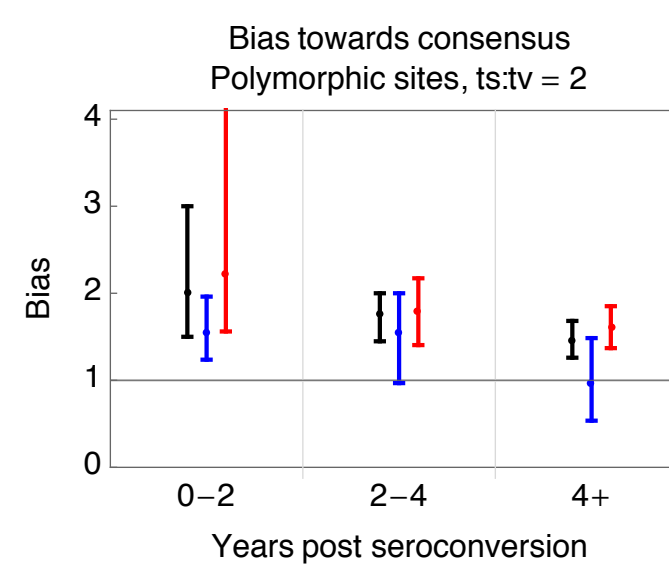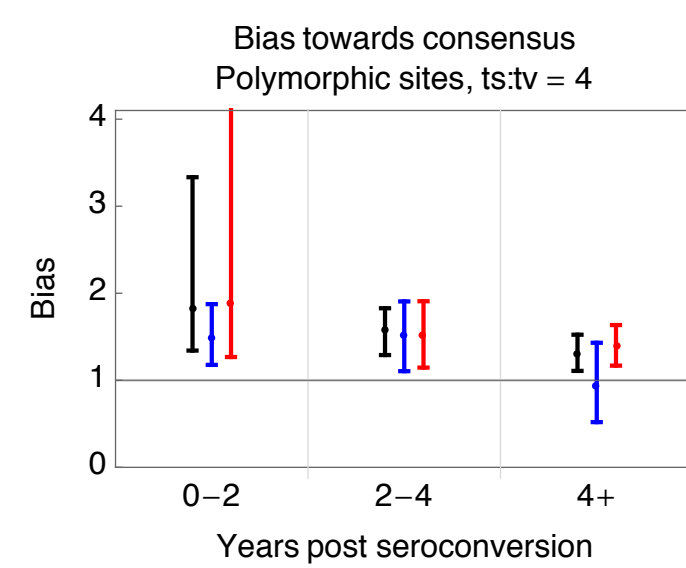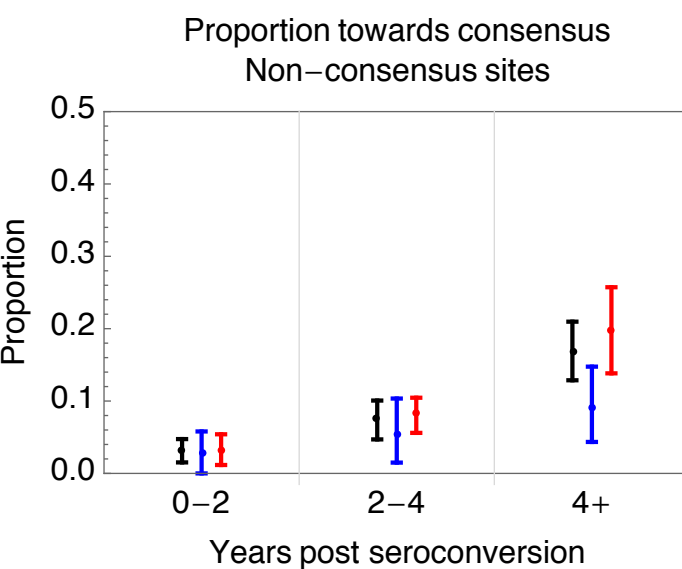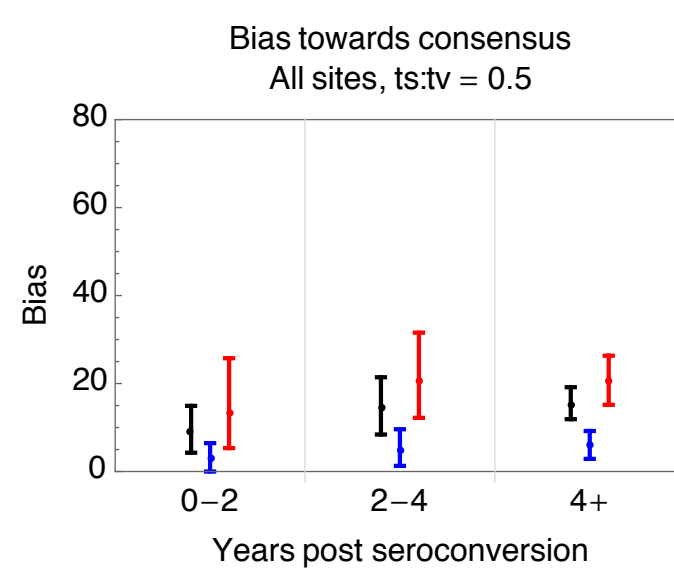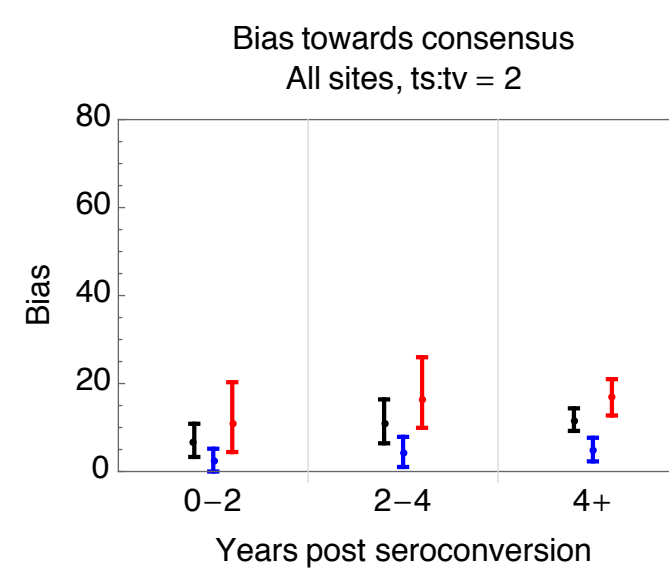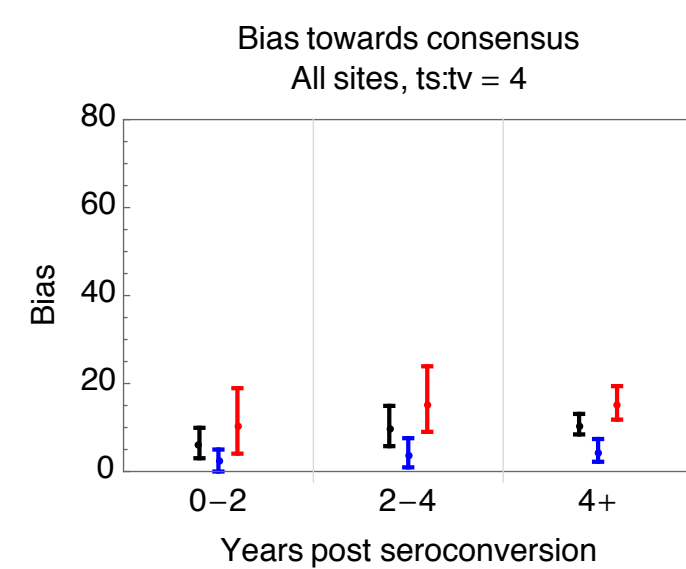

Supplement: S11 Fig — This is identical to S10 Fig, but with individuals i1, i2, i4, i9, i12, i14, i20, i25 and i34 removed since they show high diversity in the p24 gene region at the first sampling time point, indicative of infection by multiple variants from the same donor individual. In addition, i24 was also removed, due to very high diversity in gp41. In all cases, the error bars give the 5 and 95 percentiles from 10,000 bootstraps of the individual data. Black, all changes; Blue, synonymous changes; Red, nonsynonymous changes. (PDF) [file ppat.1007167.s011.pdf]

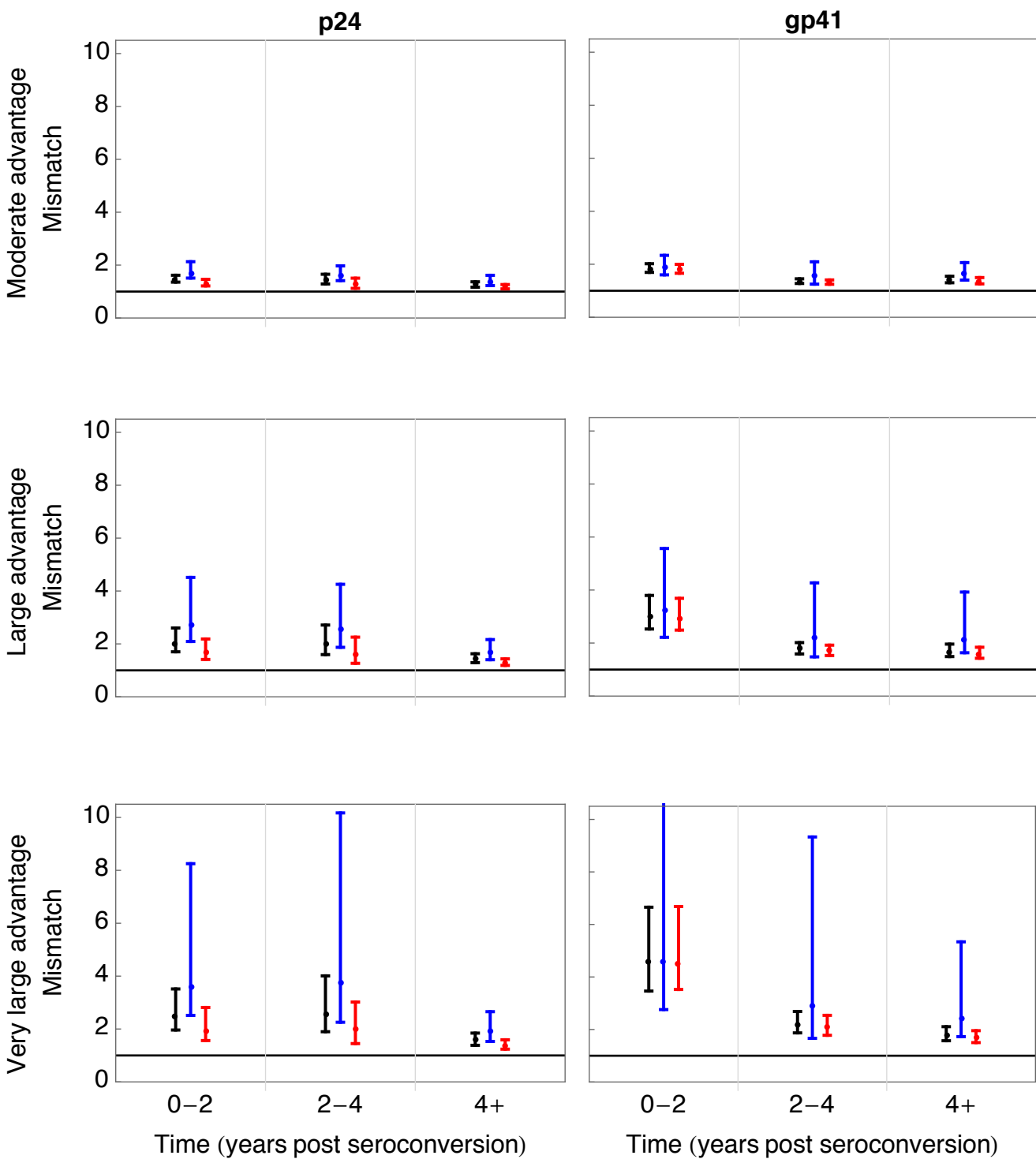

Supplement: S12 Fig — This is identical to Fig 4, but with individuals i1, i2, i4, i9, i12, i14, i20, i25 and i34 removed since they show high diversity in the p24 gene region at the first sampling time point, indicative of infection by multiple variants from the same donor individual. In addition, i24 was also removed, due to very high diversity in gp41. The error bars give the 5th and 95th percentiles from bootstrapping over the individuals 100,000 times. Black, all mutations; Blue, only synonymous mutations are considered when calculating the expected mismatch; Red, only nonsynonymous mutations are considered when calculating the expected mismatch (see Methods). (PDF) [file ppat.1007167.s012.pdf]
