# Supplementary material for: Evolution of HIV-1 within untreated individuals and at the population scale in Uganda
Source: PLoS Pathog. 2018 Jul 27;14(7):e1007167. doi: 10.1371/journal.ppat.1007167 (PMC6082572; doi:10.1371/journal.ppat.1007167)
Supplement: S6 Fig — (PDF) [file ppat.1007167.s006.pdf]

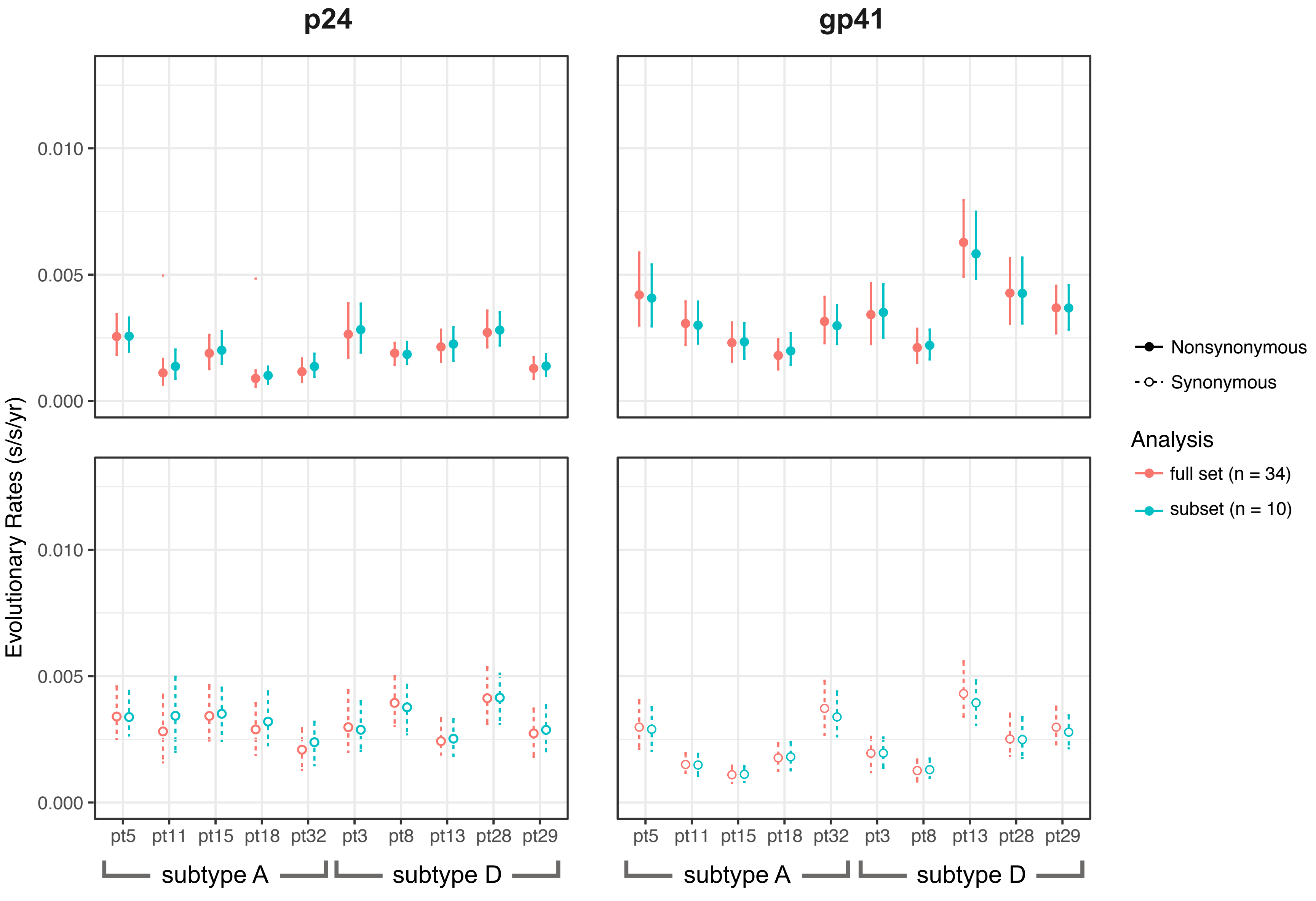

p24
gp41
0.010
0.005
0.000
Evolutionary Rates (s/s/yr)
pt5
pt11
pt15
pt18
pt32
pt3
pt8
pt13
pt28
pt29
subtype A
subtype D
Nonsynonymous
Synonymous
Analysis
full set (n = 34)
subset (n = 10)
