# Supplementary material for: Evolution of HIV-1 within untreated individuals and at the population scale in Uganda
Source: PLoS Pathog. 2018 Jul 27;14(7):e1007167. doi: 10.1371/journal.ppat.1007167 (PMC6082572; doi:10.1371/journal.ppat.1007167)
Supplement: S1 Table — (DOCX) [file ppat.1007167.s013.docx]

| **Gene** | **Substitution** | **Subtype** | **Evolutionary rate (x10^-3^ s/s/yr) ^a^** | **Ratio** |
| --- | --- | --- | --- | --- |
| Within-host evolutionary rates | | | | |
| gag | N | A | 1.68 ± 1.28 | 2.74 ± 2.08 |
| gag | S | A | 2.88 ± 8.67 | 3.79 ± 1.14 |
| gp41 | N | A | 4.21 ± 1.71 | 4.86 ± 1.97 |
| gp41 | S | A | 2.39 ± 0.80 | 3.99 ± 1.34 |
| gag | N | C | 1.04 | 8.46* |
| gag | S | C | 1.44 | 7.35* |
| gp41 | N | C | 1.23 | 3.07* |
| gp41 | S | C | 5.22 | 2.92* |
| gag | N | D | 2.01 ± 1.34 | 8.86 ± 5.92 |
| gag | S | D | 3.11 ± 0.86 | 5.39 ± 1.38 |
| gp41 | N | D | 4.40 ± 1.78 | 4.40 ± 1.78 |
| gp41 | S | D | 3.03 ± 1.19 | 4.91 ± 1.93 |
|  |  |  |  |  |
| Between-host evolutionary rates | | | | |
| gag | N | A | 0.57 ± 0.040 | - |
| gag | S | A | 0.71 ± 0.047 | - |
| gp41 | N | A | 1.04 ± 0.072 | - |
| gp41 | S | A | 0.72 ± 0.049 | - |
| gag | N | C | 0.49 ± 0.058 | - |
| gag | S | C | 0.72 ± 0.038 | - |
| gp41 | N | C | 1.44 ± 0.126 | - |
| gp41 | S | C | 0.64 ± 0.057 | - |
| gag | N | D | 0.13 ± 0.009 | - |
| gag | S | D | 0.32 ± 0.018 | - |
| gp41 | N | D | 0.98 ± 0.046 | - |
| gp41 | S | D | 0.61 ± 0.028 | - |
|  |  |  |  |  |

Table S1: Within-host and between-host evolutionary rates for subtypes A, C, and D HIV-1 infections.

Footnotes

^a^ This statistic corresponds to the mean evolutionary rate per subtype.

*Standard deviations are not shown for subtype C since only one patient sample was available per gene region.
