# Supplementary material for: Evolution of HIV-1 within untreated individuals and at the population scale in Uganda
Source: PLoS Pathog. 2018 Jul 27;14(7):e1007167. doi: 10.1371/journal.ppat.1007167 (PMC6082572; doi:10.1371/journal.ppat.1007167)
Supplement: S1 Text — (DOCX) [file ppat.1007167.s016.docx]

**Evolution of HIV-1 within untreated individuals and at the population scale in Uganda**

**S1 Text: Full description of the BEAST sensitivity Analyses**

Jayna Raghwani^1,2*^, Andrew D. Redd^3,4^*, Andrew F. Longosz^3^, Chieh-Hsi Wu^5^, David Serwadda^6,7^, Craig Martens^8^, Joseph Kagaayi^6^, Nelson Sewankambo^6,9^, Stephen F Porcella^8^, Mary K. Grabowski^4^, Thomas C. Quinn^3,4^, Michael A. Eller^10,11^, Leigh Anne Eller^10,11^, Fred Wabwire-Mangen^10,11^, Merlin L. Robb^10,11^, Christophe Fraser^1^, Katrina A. Lythgoe^1,2^

^1^Big Data Institute, Li Ka Shing Centre for Health Information and Discovery, Nuffield Department of Medicine, University of Oxford, Oxford, United Kingdom

^2^Department of Zoology, Peter Medawar Building, University of Oxford, Oxford, United Kingdom

^3^Laboratory of Immunoregulation, DIR, NIAID, NIH, Baltimore MD, USA

^4^Johns Hopkins Medical Institute, Johns Hopkins University, Baltimore MD, USA

^5^Department of Statistics, University of Oxford, United Kingdom

^6^Rakai Health Sciences Program, Kalisizo, Uganda

^7^School of Public Health, Makerere University, Kampala, Uganda

^8^Genomics Unit, RTS, RTB, Rocky Mountain Laboratories, DIR, NIAID, NIH, Hamilton MT, USA

^9^School of Medicine, Makerere University, Kampala, Uganda

^10^U.S. Military HIV Research Program, Walter Reed Army Institute of Research, Silver Spring, MD

^11^Henry M. Jackson Foundation for the Advancement of Military Medicine, Bethesda, MD

* [jayna.raghwani@bdi.ox.ac.uk](mailto:jayna.raghwani@bdi.ox.ac.uk)

To corroborate the within-host evolutionary estimates using the renaissance counting method and hierarchical phylogenetic model, we performed three sets of auxiliary analyses in BEAST for a subset of ten individuals consisting of five pure subtype A and five pure subtype D infections, i.e. for a given HIV-1 infection, p24 and gp41 gene regions corresponded to the same subtype. Furthermore, based on mean diversity at the first time point in the p24 gene region, these individuals were unlikely to have been infected by multiple, genetically distinct strains.

First, we re-estimated the within-host evolutionary rates for the subset of ten individuals using the same evolutionary model parameters employed in the original analysis based on the full set of 34 individuals. There was strong agreement in the rate estimates based on the subset of ten individuals and the full set of 34 individuals (Figure S6). This agreement indicates that individuals who were probably infected with multiple variants of HIV-1 have not influenced the estimates of evolutionary rates in individuals that were infected with a single HIV-1 variant, and further corroborates that estimates based on a smaller subset of individuals are not biased.

Second, we employed a full codon substitution model as outlined in [39] to estimate the nonsynonymous and synonymous substitution rates, along with a strict molecular clock and constant tree prior (consistent with our original analysis). Although for a few individuals a discernible difference in the within-host evolutionary rate was observed between the two substitution models, in general, there was a good correspondence in the rates of evolution for both gene regions for nonsynonymous and synonymous substitutions, as indicated by the large overlap in the 95% credible intervals (Figure S7). In particular, when interpreting these results together with divergence estimates (Figure S2), it appears that using the full codon substitution model can overestimate the nonsynonymous substitution rate (e.g. i11), whereas using the renaissance counting method can overestimate the synonymous substitution rate (e.g. i5, i13, and i32).

Last, we investigated the effect of the hierarchical phylogenetic model on the within-host evolutionary rate estimates. In the original analysis, noninformative priors were chosen for the clock rate hierarchical model; meaning that prior distribution had a large variance, enabling the posterior rate estimates to be predominantly informed by the within-host sequence data. We explored the impact of using more informative priors (or hyperpriors) on the precision parameter in the clock rate hierarchical model (clock HPM). Because the hyperprior follows a gamma distribution in the original analysis (defined by shape and scale parameters), a more informative prior can be obtained by reducing the scale parameter, which corresponds to a smaller variance in the prior distribution. Specifically, we re-ran the BEAST analysis with two different gamma hyperpriors on the precision parameter of the clock HPM, which were defined, respectively, by scale parameters of 10 and 100. In the original analysis, we used a scale parameter of 1000. The results from this analysis are summarized in Figure S8, which illustrates that the posterior distributions of the evolutionary rate are very similar across the different hyperpriors. Consequently, this indicates that the evolutionary rate estimates in our study are largely informed by sequence data, with the hyperprior having minimal effect.
